# Supplementary material for: Primary health care preparedness to integrate diabetes care in Blantyre, Malawi: A mixed methods study
Source: PLoS One. 2024 May 21;19(5):e0303030. doi: 10.1371/journal.pone.0303030 (PMC11108178; doi:10.1371/journal.pone.0303030)
Supplement: S1 File — (DOCX) [file pone.0303030.s001.docx]

**Non-Communicable Diseases Health Facility Assessment Tool**

**College of Medicine,**

**UNC Project Lilongwe, Malawi**

**May 2019**

**Background:** Non-communicable diseases (NCDs) are currently the leading killer diseases globally, and are on the rise. Noncommunicable diseases (NCDs) kill 41 million people each year, equivalent to 71% of all deaths globally. Each year, 15 million people die from NCD between the ages of 30 and 69 years; over 85% of these “premature” deaths occur in low- and middle-income countries including Malawi. The major NCDs in Malawi are diabetes mellitus, hypertension, cardiovascular disease, and some cancers, while others include renal disease, and chronic obstructive pulmonary disease (COPD) are also common. Among the challenges of NCD control in Malawi are the lack of baseline data on prevalence and risk factors, and the inadequate capacity of the existing health system to provide quality NCDs services.

The Malawi Ministry of Health (MoH) prioritizes NCDs especially in regard to prevention, early diagnosis, and proper management. In partnership with a number of stakeholders and clinical management of NCDs (with emphasis on diabetes) at the different levels of health care. A Needs Assessment is therefore essential to identify the gaps and plan appropriate interventions.

**Purpose:** To assess the Human and Infrastructural Capacity Needs Assessment for NCD Response at Health Facility Level.

**Overview:** There are **10** main sections of the tool.

They include: **(A)** Visit Information, **(B)** Health Facility Profile, **(C)** Human Resource and Skills Profile, **(D)** Facility-Based NCD Prevalence, **(E)** Clinics and Services, **(F)** Equipment, **(G)** Medicines & Sundries, **(H)** Laboratory, **(I)** Costs Related to NCDs, and **(J)** Referral System.

**Instructions:**

- For a **Yes/No** question (e.g. “Is there a pharmacy in your facility?”), please mark “**YES**” as “**Y**” and **“NO”** as “**N**” in the box provided.
- If the question asks for the “Number” (e.g. “How many weighing scales are there?”), please simply write the number of items in the appropriate box.
- Other questions will show the numbers that correspond to specific answers.
- If the question does not apply, please write “**not applicable**” or “**N/A**”.

**Abbreviations:**

- **NCD:** Non-Communicable Disease
- **DM:** Diabetes Mellitus
- **HT:** Hypertension
- **COPD:** Chronic Obstructive Pulmonary Disease
- **Cardio:** Cardiology

**A. VISIT INFORMATION**

|  |  | |  |
| --- | --- | --- | --- |
| A1 | Date of visit (dd/mm/yy) | |  |
| A2 | Officers visiting the Hospital | | 1) |
|  |  |  | 2) |
|  |  |  | 3) |
| A3 | Personnel interviewed^^[[1]](#footnote-1)^^ | | |
|  | 1 | Title/Qualification |  |
|  |  | NCD involvement |  |
|  |  | Contact |  |
|  | 2 | Name |  |
|  |  | Title/Qualification |  |
|  |  | NCD involvement |  |
|  |  | Contact |  |
|  | 3 | Name |  |
|  |  | Title/Qualification |  |
|  |  | NCD involvement |  |
|  |  | Contact |  |
|  | 4 | Name |  |
|  |  | Title/Qualification |  |
|  |  | NCD involvement |  |
|  |  | Contact |  |

**B. HEALTH FACILITY PROFILE**

|  | | | | | | |
| --- | --- | --- | --- | --- | --- | --- |
| B1 | Name of health facility |  | | | | |
| B2 | City/town |  | | | | |
| B3 | Full address of health facility |  | | | | |
| B4 | Is the health facility urban or rural? | | Urban  Rural | 🞎  🞎 | Public  Private | 🞎  🞎 |
| B5 | Health facility category:  Central Hospital– **1**; District Hospital – **2**; Health Centre – **3** | | | | | 🞎 |

**C. HUMAN RESOURCE AND SKILLS PROFILE**

|  | | | | |
| --- | --- | --- | --- | --- |
| **Are the following personnel in the facility, and if so, how many of each specialty/position?** | | | | |
|  | **Specialty/Position** | **Yes/No** | **Number** | **Additional Skills^^[[2]](#footnote-2)^^** |
| C1 | Cardiologist |  |  |  |
| C2 | Clinical Officer(s) |  |  |  |
| C3 | Community Health Worker(s) |  |  |  |
| C4 | Endocrinologist/Diabetologist |  |  |  |
| C5 | Foot care specialist |  |  |  |
| C6 | General surgeon |  |  |  |
| C7 | Laboratory technician(s) |  |  |  |
| C8 | Laboratory technologist(s) |  |  |  |
| C9 | Medical Officer(s) |  |  |  |
| C10 | Social workers |  |  |  |
| C11 | Midwives |  |  |  |
| C12 | NCD Health Educator |  |  |  |
| C13 | Neurologist |  |  |  |
| C14 | Nurse(s) – general |  |  |  |
| C15 | Nurse(s) – diabetic |  |  |  |
| C16 | Nurse(s) – psychiatric |  |  |  |
| C17 | Nutritionist(s) |  |  |  |
| C18 | Obstetrician/Gynecologist |  |  |  |
| C19 | Ophthalmologist |  |  |  |
| C20 | Pediatricians |  |  |  |
| C21 | Physiotherapist |  |  |  |
| C22 | Psychiatrist |  |  |  |
| C23 | Pulmonologist (or Chest physician) |  |  |  |
| C24 | Radiologist(s) |  |  |  |
| C25 | Radiology technician(s) |  |  |  |
| C26 | Specialist Physician |  |  |  |
| C27 | Vascular Surgeon |  |  |  |
| C28 | Other, specify: |  |  |  |

**C. HUMAN RESOURCE AND SKILLS PROFILE**

|  | | | | |
| --- | --- | --- | --- | --- |
| **Are health workers personnel trained on non-communicable disease (NCD) management?** | | | | |
|  | **Specialty/Position** | **Yes, regularly trained** | **Yes, trained one-time** | **No, not trained on NCD management** |
| C1 | Cardiologist |  |  |  |
| C2 | Clinical Officer(s) |  |  |  |
| C3 | Community Health Worker(s) |  |  |  |
| C4 | Endocrinologist/Diabetologist |  |  |  |
| C5 | Foot care specialist |  |  |  |
| C6 | General surgeon |  |  |  |
| C7 | Laboratory technician(s) |  |  |  |
| C8 | Laboratory technologist(s) |  |  |  |
| C9 | Medical Officer(s) |  |  |  |
| C10 | Social workers |  |  |  |
| C11 | Midwives |  |  |  |
| C12 | NCD Health Educator |  |  |  |
| C13 | Neurologist |  |  |  |
| C14 | Nurse(s) – general |  |  |  |
| C15 | Nurse(s) – diabetic |  |  |  |
| C16 | Nurse(s) – psychiatric |  |  |  |
| C17 | Nutritionist(s) |  |  |  |
| C18 | Obstetrician/Gynecologist |  |  |  |
| C19 | Ophthalmologist |  |  |  |
| C20 | Pediatricians |  |  |  |
| C21 | Physiotherapist |  |  |  |
| C22 | Psychiatrist |  |  |  |
| C23 | Pulmonologist (or Chest physician) |  |  |  |
| C24 | Radiologist(s) |  |  |  |
| C25 | Radiology technician(s) |  |  |  |
| C26 | Specialist Physician |  |  |  |
| C27 | Vascular Surgeon |  |  |  |
| C28 | Other, specify: |  |  |  |

**D. FACILITY-BASED NCDs PREVALENCE**

|  | **number of cases IN LAST FINANCIAL YEAR^^[[3]](#footnote-3)^^** | | | | **Number** |
| --- | --- | --- | --- | --- | --- |
| D1 | Estimated number of overall patients in your facility^^[[4]](#footnote-4)^^ | | | |  |
| D2 | Total number of diabetes cases | | | |  |
| D3 | Adults | | | |  |
|  |  | Male | | |  |
|  |  | Female | | |  |
|  |  |  | | Pregnant women |  |
|  |  |  | | Non-pregnant |  |
| D4 | Children | | | |  |
|  |  | | Male | |  |
|  |  | | Female | |  |
| D5 | Total number of hypertension cases | | | |  |
| D6 | Adults | | | |  |
|  |  | Male | | |  |
|  |  | Female | | |  |
|  |  |  | | Pregnant women |  |
|  |  |  | | Non-pregnant |  |
| D7 | Children | | | |  |
|  |  | | Male | |  |
|  |  | | Female | |  |
| D8 | Total number of heart disease cases | | | |  |
| D11 | Adults | | | |  |
|  |  | Male | | |  |
|  |  | Female | | |  |
|  |  |  | | Pregnant women |  |
|  |  |  | | Non-pregnant |  |
| D12 | Children | | | |  |
|  |  | | Male | |  |
|  |  | | Female | |  |
| D13 | Total number of asthma cases | | | |  |
| D14 | Adults | | | |  |
|  |  | Male | | |  |
|  |  | Female | | |  |
| D13 | Children | | | |  |
|  |  | | Male | |  |
|  |  | | Female | |  |
| D14 | Total number of other COPD cases^^[[5]](#footnote-5)^^ | | | |  |
| D15 | Adults | | | |  |
|  |  | Male | | |  |
|  |  | Female | | |  |
| D16 | Children | | | |  |
|  |  | | Male | |  |
|  |  | | Female | |  |
| D17 | Total number of stroke cases | | | |  |
| D18 | Adults | | | |  |
|  |  | Male | | |  |
|  |  | Female | | |  |
| D19 | Total number of renal disease cases | | | |  |
| D20 | Adults | | | |  |
|  |  | Male | | |  |
|  |  | Female | | |  |

**E. CLINICS AND SERVICES**

| **Clinics** | | | | | | | | | |
| --- | --- | --- | --- | --- | --- | --- | --- | --- | --- |
|  | | **DM** | **HT** | **Cardio** | **COPD/Asthma** | **Renal** | **OB/GY** | **Ped** | **HIV** |
| E1 | Do you have a clinic (NCD)? (**Y/N**) |  |  |  |  |  |  |  |  |
| E2 | Do you have a separate (NCD) clinic room? (**Y/N**) |  |  |  |  |  |  |  |  |
| E3 | How often is the clinic held? |  |  |  |  |  |  |  |  |
| E4 | When is the clinic held? |  |  |  |  |  |  |  |  |
| E5 | Is there regular patient review? (**Y/N**) |  |  |  |  |  |  |  |  |
| E6 | If yes, how often is each patient reviewed? |  |  |  |  |  |  |  |  |
| E7 | Does the clinic conduct performance audits? (**Y/N**) |  |  |  |  |  |  |  |  |
| E8 | If yes, specify.^^[[6]](#footnote-6)^^ |  |  |  |  |  |  |  |  |

| **Services (DEPERTMENT-IN-CHARGE)** | | | | | | | | | | | |
| --- | --- | --- | --- | --- | --- | --- | --- | --- | --- | --- | --- |
|  |  | | **DM** | **HT** | **Cardio** | **COPD/Asthma** | **Renal** | **OB/GY** | **Ped** | **HIV** | |
|  | **Are the following services offered?** | | **Y/N** | **Y/N** | **Y/N** | **Y/N** | **Y/N** | **Y/N** | **Y/N** | **Y/N** | |
| E9 | Blood Pressure measurement | |  |  |  |  |  |  |  |  | |
| E10 | Weight | |  |  |  |  |  |  |  |  | |
| E11 | Height | |  |  |  |  |  |  |  |  | |
| E12 | BMI calculation | |  |  |  |  |  |  |  |  | |
| E13 | Waist: Hip ratio | |  |  |  |  |  |  |  |  | |
| E14 | Blood glucose | |  |  |  |  |  |  |  |  | |
| E15 | Oral Glucose Tolerance Test (OGTT) | |  |  |  |  |  |  |  |  | |
| E16 | Blood lipids | |  |  |  |  |  |  |  |  | |
| E17 | Urinalysis | |  |  |  |  |  |  |  |  | |
| E18 | Urine protein | |  |  |  |  |  |  |  |  | |
| E19 | Urine ketones | |  |  |  |  |  |  |  |  | |
| E20 | Eye examination | |  |  |  |  |  |  |  |  |  |
| E21 | Individual patient NCD education | |  |  |  |  |  |  |  |  |  |
| E22 | Group NCD education | |  |  |  |  |  |  |  |  |  |
| E23 | Foot care for Diabetic patients | |  |  |  |  |  |  |  |  |  |
| E24 | Nutrition advice for all patients | |  |  |  |  |  |  |  |  |  |
| E25 | Radiotherapy | |  |  |  |  |  |  |  |  |  |
| E26 | Physiotherapy | |  |  |  |  |  |  |  |  |  |
| E27 | Record of family history of NCDs | |  |  |  |  |  |  |  |  |  |
| E28 | Provision of NCDs IEC materials | |  |  |  |  |  |  |  |  |  |
| E29 | Self-management support^^[[7]](#footnote-7)^^ | |  |  |  |  |  |  |  |  |  |
| E30 | Peer/social support linkage^^[[8]](#footnote-8)^^ | |  |  |  |  |  |  |  |  |  |
| **Guidelines (DEPARTMENT IN-CHARGE)** | | | | | | | | | | |  |
|  | |  | **DM** | **HT** | **Cardio** | **COPD** | **Renal** | **OB/GY** | **Ped** | **HIV** |  |
|  | | **Are the following guidelines used?** | **Y/N** | **Y/N** | **Y/N** | **Y/N** | **Y/N** | **Y/N** | **Y/N** | **Y/N** |  |
| E41 | | Diabetes management |  |  |  |  |  |  |  |  |  |
| E42 | | Hypertension management |  |  |  |  |  |  |  |  |  |
| E43 | | Hyperlipidemia management |  |  |  |  |  |  |  |  |  |
| E44 | | Tobacco screening & treatment |  |  |  |  |  |  |  |  |  |
| E45 | | Alcohol screening & treatment |  |  |  |  |  |  |  |  |  |
| E48 | | Asthma/COPD management |  |  |  |  |  |  |  |  |  |
| E50 | | Palliative care |  |  |  |  |  |  |  |  |  |

| **HIV clinic** | | |
| --- | --- | --- |
| **Number of known HIV patients** | | |
| E39 | Total number of active patients with HIV in the last financial year^^[[9]](#footnote-9)^^ | Male:  Female: |
| E40 | Number of new cases in last 3 financial years | 2015-16: |
|  |  | 2017-18: |
|  |  | 2018-19: |
| E42 | Number of active HIV patients with diabetes |  |
| E43 | Number of active HIV patients with hypertension |  |
| E44 | Number of active HIV patients with CVD |  |

**F. EQUIPMENT**

|  | | | | | **DM** | | **HT** | | **Cardio** | | **COPD** | | **Renal** | | | **OB/GY** | | | **Ped** | | **HIV** | |
| --- | --- | --- | --- | --- | --- | --- | --- | --- | --- | --- | --- | --- | --- | --- | --- | --- | --- | --- | --- | --- | --- | --- |
| **Are the following equipment’s available in the all areas where NCD clinics are provided? (Y/N and number)** | | | | | **Y/N** | **#** | **Y/N** | **#** | **Y/N** | **#** | **Y/N** | **#** | **Y/N** | **#** | | **Y/N** | **#** | | **Y/N** | **#** | **Y/N** | **#** |
| F1 | Clinicians table | | | |  |  |  |  |  |  |  |  |  |  | |  |  | |  |  |  |  |
| F2 | Nurses station/table | | | |  |  |  |  |  |  |  |  |  |  | |  |  | |  |  |  |  |
| F3 | Hand washing basin/sink with soap | | | |  |  |  |  |  |  |  |  |  |  | |  |  | |  |  |  |  |
| F4 | Patient files | | | |  |  |  |  |  |  |  |  |  |  | |  |  | |  |  |  |  |
| F5 | File cabinet/cupboard/storage space | | | |  |  |  |  |  |  |  |  |  |  | |  |  | |  |  |  |  |
| F6 | NCD register: for new cases only | | | |  |  |  |  |  |  |  |  |  |  | |  |  | |  |  |  |  |
| F7 | NCD register: for follow-up cases only | | | |  |  |  |  |  |  |  |  |  |  | |  |  | |  |  |  |  |
| F8 | NCD register: for admission only | | | |  |  |  |  |  |  |  |  |  |  | |  |  | |  |  |  |  |
| F9 | BP machine: Mercury sphygmoman-ometer | | | Number present |  |  |  |  |  |  |  |  |  |  | |  |  | |  |  |  |  |
| F10 |  |  |  | Number functional |  |  |  |  |  |  |  |  |  |  | |  |  | |  |  |  |  |
| F11 |  |  |  | # Calibrated |  |  |  |  |  |  |  |  |  |  | |  |  | |  |  |  |  |
| F12 | BP machine: Aneroid | | | Number present |  |  |  |  |  |  |  |  |  |  | |  |  | |  |  |  |  |
| F13 |  |  |  | Number functional |  |  |  |  |  |  |  |  |  |  | |  |  | |  |  |  |  |
| F14 |  |  |  | # Calibrated |  |  |  |  |  |  |  |  |  |  | |  |  | |  |  |  |  |
| F15 | BP machine: Automated | | | Number present |  |  |  |  |  |  |  |  |  |  | |  |  | |  |  |  |  |
| F16 |  |  |  | Number functional |  |  |  |  |  |  |  |  |  | |  |  | |  |  |  |  |  |
| F17 |  |  |  | # Calibrated |  |  |  |  |  |  |  |  |  | |  |  | |  |  |  |  |  |
| F18 | BP cuffs: Standard (25 cm x 12 cm) | | | |  |  |  |  |  |  |  |  |  | |  |  | |  |  |  |  |  |
| F19 | BP cuffs: Alternate (36 cm x 12 cm) | | | |  |  |  |  |  |  |  |  |  | |  |  | |  |  |  |  |  |
| F20 | BP cuffs: Paediatric | | | |  |  |  |  |  |  |  |  |  | |  |  | |  |  |  |  |  |
| F21 | Stethoscope | | Number present | |  |  |  |  |  |  |  |  |  | |  |  | |  |  |  |  |  |
| F22 |  |  | Number functional | |  |  |  |  |  |  |  |  |  | |  |  | |  |  |  |  |  |
| F23 | Blood  Glucose meter | | Number present | |  |  |  |  |  |  |  |  |  | |  |  | |  |  |  |  |  |
| F24 |  |  | Number functional | |  |  |  |  |  |  |  |  |  | |  |  | |  |  |  |  |  |
| F25 |  |  | # Calibrated correctly | |  |  |  |  |  |  |  |  |  | |  |  | |  |  |  |  |  |
| F26 |  |  | Cost of strips per unit | |  |  |  |  |  |  |  |  |  | |  |  | |  |  |  |  |  |
| F27 | Urine testing strips | | Multiple test | |  |  |  |  |  |  |  |  |  | |  |  | |  |  |  |  |  |
| F29 |  |  | Availability? Always – **1**; Sometimes – **2**; Never – **0** | |  |  |  |  |  |  |  |  |  | |  |  | |  |  |  |  |  |
| F30 | Weighing scales | | # Bathroom type | |  |  |  |  |  |  |  |  |  | |  |  | |  |  |  |  |  |
| F31 |  |  | # Hospital type | |  |  |  |  |  |  |  |  |  | |  |  | |  |  |  |  |  |
| F32 | Height meters | | Number present | |  |  |  |  |  |  |  |  |  | |  |  | |  |  |  |  |  |
| F33 |  |  | # Calibrated correctly | |  |  |  |  |  |  |  |  |  | |  |  | |  |  |  |  |  |
| F34 | Ophthalmo-scope | | Number present | |  |  |  |  |  |  |  |  |  | |  |  | |  |  |  |  |  |
| F35 |  |  | Number functional | |  |  |  |  |  |  |  |  |  | |  |  | |  |  |  |  |  |
| F36 | Snellen charts (visual acuity exam) | | # Handheld type | |  |  |  |  |  |  |  |  |  | |  |  | |  |  |  |  |  |
| F37 |  |  | # Distance type | |  |  |  |  |  |  |  |  |  | |  |  | |  |  |  |  |  |
|  | **Other equipment** | | | |  |  |  |  |  |  |  |  |  |  | |  |  | |  |  |  |  |
| F38 | BMI chart | | | |  |  |  |  |  |  |  |  |  |  | |  |  | |  |  |  |  |
| F39 | Measuring tapes | | | |  |  |  |  |  |  |  |  |  |  | |  |  | |  |  |  |  |
| F40 | Tuning forks | | | |  |  |  |  |  |  |  |  |  |  | |  |  | |  |  |  |  |
| F41 | Monofilament | | | |  |  |  |  |  |  |  |  |  |  | |  |  | |  |  |  |  |
| F42 | Spirometer | | | |  |  |  |  |  |  |  |  |  |  | |  |  | |  |  |  |  |
| F43 | Spacers for inhalers | | | |  |  |  |  |  |  |  |  |  |  | |  |  | |  |  |  |  |
| F44 | Nebuliser | | | |  |  |  |  |  |  |  |  |  |  | |  |  | |  |  |  |  |
| F45 | Thermometers | | | |  |  |  |  |  |  |  |  |  |  | |  |  | |  |  |  |  |
| F46 | Bronchoscope | | | |  |  |  |  |  |  |  |  |  |  | |  |  | |  |  |  |  |
| F47 | Emergency Trolley/Tray | | | |  |  |  |  |  |  |  |  |  |  | |  |  | |  |  |  |  |
| F48 | Physiotherapy equipment | | | |  |  |  |  |  |  |  |  |  |  | |  |  | |  |  |  |  |
| F49 | Other  (Specify |  | | |  |  |  |  |  |  |  |  |  |  | |  |  | |  |  |  |  |
|  |  |  | | |  |  |  |  |  |  |  |  |  |  | |  |  | |  |  |  |  |
|  | | | | | | | | | | | | | | | | | | | | | | |
|  | Maintenance | | | | **DM** | | **HT** | | **Cardio** | | **COPD** | | **Renal** | | | **OB/GY** | | | **Ped** | | **HIV** | |
|  |  | | | | **Y/N** | **#** | **Y/N** | **#** | **Y/N** | **#** | **Y/N** | **#** | **Y/N** | **#** | | **Y/N** | **#** | | **Y/N** | **#** | **Y/N** | **#** |
| F50 | Is there a written equipment maintenance plan? | | | |  |  |  |  |  |  |  |  |  |  | |  |  | |  |  |  |  |
| F51 | If yes, is the plan implemented? | | | |  |  |  |  |  |  |  |  |  |  | |  |  | |  |  |  |  |
| F52 | How often are maintained? | | | | Once a year or more | | | | Less than once a year | | | | | Never | | | | | Don’t know | | | |
|  |  | | | |  |  |  |  |  |  |  |  |  |  | |  |  | |  |  |  |  |
|  | **Imaging** | | | | **Available?** | | | | | | | | | | | **Functional?** | | | | | | |
| F53 | Ultrasound scan | | | |  | | | | | | | | | | |  | | | | | | |
| F54 | Echography | | | |  | | | | | | | | | | |  | | | | | | |
| F55 | ECG monitor | | | |  | | | | | | | | | | |  | | | | | | |
| F56 | X-Rays | | | |  | | | | | | | | | | |  | | | | | | |
| F57 | Doppler | | | |  | | | | | | | | | | |  | | | | | | |
| F58 | CT-Scan | | | |  | | | | | | | | | | |  | | | | | | |
| F59 | Other, specify | | | |  | | | | | | | | | | |  | | | | | | |
|  | **Power Supply** | | | | **Available?** | | | | | | | | | | | **Functional?** | | | | | | |
| F60 | Reliable power supply | | | |  | | | | | | | | | | |  | | | | | | |
| F61 | Alternative source of power^^[[10]](#footnote-10)^^ | | | |  | | | | | | | | | | |  | | | | | | |

**G. MEDICINES AND SUNDRIES (PHARMACIST)**

| G1 | Is there a drug store (pharmacy) in your facility? (**Y/N**) | | | | | |  | | |
| --- | --- | --- | --- | --- | --- | --- | --- | --- | --- |
| **Medicines** | | | | | | | | | |
|  | | **Classes of drugs** | | **Available** | **How often are they stocked?** | | | **Was there a stock-out in the last quarter?** | **Was there a stock-out in the last financial year?** |
|  |  |  |  |  | **Every 2 mos.**  **Quarterly**  **Other** | **1**  **23** | |  |  |
|  | | **Anti-hypertensives** | |  |  | | |  |  |
| G2 | | Thiazide diuretic (e.g. Aprinox) | |  |  | | |  |  |
| G3 | | Calcium channel blocker (e.g. Nifedipine) | |  |  | | |  |  |
| G4 | | Beta-blocker (e.g. Propranolol) | |  |  | | |  |  |
| G5 | | ACE inhibitor (e.g. Captopril) | |  |  | | |  |  |
| G6 | | Others (e.g. Aldomet, Hydrallazine, Magnesium Sulphate) | |  |  | | |  |  |
|  | | **Diabetic drugs** | |  |  | | |  |  |
| G7 | | Biguanides (e.g. Metformin) | |  |  | | |  |  |
| G8 | | Sulfonylureas (e.g. Glibenclamide) | |  |  | | |  |  |
| G9 | | Thiazolidinediones (e.g. Pioglitazone) | |  |  | | |  |  |
| G10 | | Dipeptidyl peptidase-4 inhibitors | |  |  | | |  |  |
| G11 | | Alpha-glucosidase inhibitors | |  |  | | |  |  |
| G12 | | Others | |  |  | | |  |  |
| G13 | | Insulin type available | Ultra short-acting |  |  | | |  |  |
| G14 | |  | Short-acting |  |  | | |  |  |
| G15 | |  | Intermediate |  |  | | |  |  |
| G16 | |  | Long-acting |  |  | | |  |  |
| G17 | | Strength of insulin available | U 100 |  |  | | |  |  |
|  |  |  | Other |  |  | | |  |  |
| G18 | | Insulin syringes (e.g. U100) | |  |  | | |  |  |
|  | | **Asthma** | |  |  | | |  |  |
| G19 | | Short acting Beta2-agonists (e.g. salbutamol, terbutsaline) | |  |  | | |  |  |
| G20 | | Anticholinergics (e.g. ipratropium bromide) | |  |  | | |  |  |
| G21 | | Inhaler steroids (beclomethasone inhaler) | |  |  | | |  |  |
| G22 | | Oral steroids (e.g. oral predenisole) | |  |  | | |  |  |
| G23 | | Long acting inhaled beta2 agosnist (e.g. salmeterol, formterol) | |  |  | | |  |  |
| G24 | | Sustained release theophylline tablets (e.g. aminophylline) | |  |  | | |  |  |
| G25 | | Leukotriene antagonists (e.g. monterlukast, zafirlukast) | |  |  | | |  |  |
|  | | **For other diseases** | |  |  | | |  |  |
| G20 | | Statins | |  |  | | |  |  |
| G21 | | Cardiac Aspirin | |  |  | | |  |  |
| G22 | | Sulfadoxine/pyrimethamine (e.g. Fansidar) | |  |  | | |  |  |
| G23 | | Antibiotics, specify: | |  |  | | |  |  |
| G24 | | Anticoagulants | |  |  | | |  |  |
| G26 | | Other^^[[11]](#footnote-11)^^ | |  |  | | |  |  |
| **Other Questions** | | | | | | | | | |
|  | | What is the source of procurement? | | | | | | | |
| G27 | | Government | | | | |  | | |
| G28 | | Other, and specify^^[[12]](#footnote-12)^^ | | | | |  | | |
| G29 | | Do you get the types of drugs you ask for? | | | | |  | | |
| G30 | | Do you get the quantity of drugs you ask for? | | | | |  | | |
| G31 | | Do you have a refrigerator in the drug store (pharmacy)? | | | | |  | | |

**H. LABORATORY**

|  | **Question** | **Yes/NO** |
| --- | --- | --- |
| H1 | Is there a laboratory in the facility? |  |
| **Are the following lab tests done?** | | |
| H2 | Electrolytes (e.g., potassium) |  |
| H3 | Full blood count and differential |  |
| H4 | Full Urinalysis |  |
| H5 | Hb electrophoresis |  |
| H6 | HbA1c |  |
| H7 | Hemocult |  |
| H8 | Hemoglobin |  |
| H9 | Lipid Profile |  |
| H10 | Liver Function Tests |  |
| H11 | Microalbuminuria |  |
| H12 | Random Blood Sugar |  |
| H13 | Renal Function Tests |  |
| H14 | Other, specify^^[[13]](#footnote-13)^^ |  |
| H15 | Is there a functional centrifuge available? |  |
| H16 | Is there a functional microscope available? |  |
| H17 | Is there a functional refrigerator available? |  |

1. **COSTS RELATED TO NCDS^^[[14]](#footnote-14)^^**

|  | **General Item** | **Specify Item** | **Cost Estimate^^[[15]](#footnote-15)^^** |
| --- | --- | --- | --- |
| I1 | Drugs |  |  |
|  |  |  |  |
| I2 | Labs |  |  |
|  |  |  |  |
| I3 | Imaging |  |  |
|  |  |  |  |
| I4 | Other Services |  |  |
|  |  |  |  |

**J. REFERRAL SYSTEM – DISTRICTS AND HEALTH**

|  | **question** | | | | | | | | | | | **yes/no** |
| --- | --- | --- | --- | --- | --- | --- | --- | --- | --- | --- | --- | --- |
| J1 | **Health System:** Availability of protocols for management and referral of NCD patients in this facility. | | | | | | | | | | |  |
| J2 | **If yes specify:** |  | | | | | | | | | | |
|  |  |  | | | | | | | | | | |
|  |  |  | | | | | | | | | | |
| J3 | **Health System:** Availability of a referral form (or any other form of communication) to the next facility | | | | | | | | | Outgoing | |  |
|  |  |  |  |  |  |  |  |  |  | Receiving | |  |
| J4 | **Health System:** Availability of transport to the next facility | | | | | | | | | | |  |
| J5 | **Referral Practicalities:** A register exists to monitor follow-up and gather statistics on referrals | | | | | | | | | Outgoing | |  |
|  |  |  |  |  |  |  |  |  |  | Receiving | |  |
| J5 | **Can you refer patients to another facility in the event of a chronic disease emergency?** | | | | | | | | |  | |  |
| *If you answered “Yes”, go to Question J5. If you answered “No”, skip to Question J12* | | | | | | | | | | | | |
| J6 | **How many kilometers**  **(kms) from your**  **facility is the nearest**  **referral institution for**  **a medical emergency?** | |  | | | **Hrs** |  | | **Mins** | |  | **Km** |
|  |  |  |  |  |  |  |  |  |  |  |  |  |
|  |  |  |  | |  |  |  | |  |  |  |  |
|  |  |  |  |  |  |  |  |  |  |  |  |  |
|  |  | | | | | | | | | Yes | | No |
| J7 | **Have you ever wanted to refer a patient with acute, severe symptoms or emergency related to heart disease, diabetes or asthma but were unable to do so?** | | | | | | | | |  | |  |
|  |  | | | | | | | | | Why? | |  |
|  |  | | | | | | | | |  | |  |
|  |  | | | | | | | **Yes** | | No | | Don’t know |
| J8 | **Does your facility have an ambulance?** | | | | | | |  | |  | |  |
| J9 | **If the facility does not have an ambulance, can patient transfer by ambulance be arranged?** | | | | | | |  | |  | |  |
|  |  | | | Ambulance | | | | Public transport | | Private vehicle | | Commercial vehicle (e.g. taxi) |
| J10 | **What means of transport is most frequently used to transfer emergency patients at your facility (check only one)?** | | |  | | | |  | |  | |  |
|  |  | | | Others specify | | | | | | | | |
| J11 | **Approximately how long does it take to transfer a patient to the nearest referral medical institution?** | | | | | | | **Hours** | | **Minutes** | | **Days** |
|  |  | | | | | | |  | | **Yes** | | **Not** |
| J12a | **Can you refer patients with noncommunicable diseases (NCDs) for a second opinion/specialist consultation?** | | | | | | | | |  | |  |
|  | If no why? | | | | | | | | | | | |
| J12b | **If “yes”, the patients will usually be?** | | | Referred back to you for follow-up | | | | | | Followed up at the upper level (referral) facility | | |
|  |  | | |  | | | |  | |  | |  |
|  |  | | |  | | | |  | | Yes | | No. Why? |
| J12c | **Can you refer patients with noncommunicable diseases (NCDs) to the nearest referral medical institution for some additional test?** | | | | | | | | |  | |  |
|  | | | | | | | | | | | | |

**K. FINANCING AND ADMINISTRATION**

| K1 | **Do patients pay the facility for medicines?** | Yes, full payment | Yes, partial payment > Proportion paid by patient… % | | No, medicines are provided for free |
| --- | --- | --- | --- | --- | --- |
|  |  | | | | |
| K2 | **If medicines are provided for free or for partial payment, who subsidizes it?** | Central government | Local government | Private insurance | Social assistance plans |
|  |  | Other (specify | | Don’t know | |
| K3 | **Do patients pay the facility for consultations?** | Yes, full payment | Yes, partial  payment > Proportion paid by  patient… % | | No, medicines are provided for free |
|  |  | Other (specify | | Don’t know | |
| K4 | **If consultations are provided for free or for partial payment, who subsidizes it?** | Central government | Local government | Private insurance | Social assistance plans |
| K5 | **Do patients pay the facility for diagnostic tests?** | Yes, full payment | Yes, partial  payment > Proportion paid by  patient… % | | No, medicines are provided for free |
|  |  | Other (specify | | Don’t know | |
| K6 | **If diagnostic tests are provided for free or for partial payment, who subsidizes it?** | Central government | Local government | Private insurance | Social assistance plans |
|  |  |  |  |  |  |

1. Personnel interviewed should be from any of the following areas: Administration, Pharmacy, Clinicians in NCD clinics (including nurses), Records, and/or Laboratory, Physiotherapist. [↑](#footnote-ref-1)
2. E.g. Foot care, nutrition, and any other skill. Please list skill and then number of personnel with the specified skill. For example: Foot care (2). [↑](#footnote-ref-2)
3. Please obtain actual number of patients from register. [↑](#footnote-ref-3)
4. Both inpatient (admission) and outpatient. [↑](#footnote-ref-4)
5. COPD – defined as progressive symptoms of cough, and/or sputum production, and/or dyspnea for more than 3 months where asthma and infectious causes have been excluded. [↑](#footnote-ref-5)
6. e.g. loss to follow-up, deaths, availability of drugs, etc. [↑](#footnote-ref-6)
7. e.g. Lifestyle modification [↑](#footnote-ref-7)
8. Including expert patients [↑](#footnote-ref-8)
9. An “active patient” has attended the HIV clinic within the last 6 months. [↑](#footnote-ref-9)
10. e.g. standby generator, solar, etc. [↑](#footnote-ref-10)
11. Include renal, and other drugs. [↑](#footnote-ref-11)
12. e.g. Special fund for health, independent wholesaler, gift from a philanthropic body [↑](#footnote-ref-12)
13. e.g. Carcinoembryonic Antigen [↑](#footnote-ref-13)
14. Costs for patients related to NCDs. [↑](#footnote-ref-14)
15. Estimate cost for 1 month’s supply of drugs and 1 visit for labs, imaging, and other services. [↑](#footnote-ref-15)
