# Supplementary material for: Primary health care preparedness to integrate diabetes care in Blantyre, Malawi: A mixed methods study
Source: PLoS One. 2024 May 21;19(5):e0303030. doi: 10.1371/journal.pone.0303030 (PMC11108178; doi:10.1371/journal.pone.0303030)
Supplement: S2 File — (DOCX) [file pone.0303030.s002.docx]

**INDEPTH INTERVIEW GUIDE: HEALTH STAFF**

General information of interviewee

| Full name: | Sex: | Male | Female | Age: |
| --- | --- | --- | --- | --- |

| Position | |
| --- | --- |
| Director/DHO |  |
| Doctor |  |
| Nurse |  |
| Pharmacist |  |
| Lab technology/technician |  |
| Physiotherapist |  |

| Level Hospital | |
| --- | --- |
| Central Hospital |  |
| District Hospital |  |
| Health center |  |

**Introduction**

Thank you for agreeing to spend time to answer more detailed questions about Non-communicable diseases (NCDs). This interview will not take more than one hour and we will be discussing the role of Central Medical stores in the management of NCDs in Malawi. I will be taping the session because I don’t want to miss any of your comments.

1. Could you please introduce yourself and your role/profession?
   - When did you graduate? How long have you been working here?
   - Tell me about your background.
2. How would you describe an ordinary working day – what do you do (ask for details and examples)?
   - How do you think your training meets your needs at work?
3. How do you define NCDs? Please list NCDs you know. Which of these NCDs would you rate as the biggest problem in Malawi and your catchment area? Please explain why?

Probe: How big is diabetes in your catchment area?

1. If a patient has the one or more of the conditions (NCD) you have just mentioned – what health services/primary health services do you think should be available at a health facility? And what NCD services are actually implemented at this health facility? Please describe.

Probe: what services should be there for diabetes? What services are actually there for diabetes?

1. To what extent has each of the NCD services been implemented in your facility? What guidelines are available for these conditions, if any?
   - What worked well to meet the needs of your target population? Please elaborate.
   - How do you see the accessibility of these NCD services? Please explain.
   - What are your thoughts about the extent of their coverage? And efficiency? Please explain.
   - What could be improved? Please explain why.

Probe: how about for diabetes?

1. What strategies and policies related to primary healthcare services for NCDs are you aware of?
   - How do they relate to your current work on NCD patients?
   - What strategies and policies should be added to improve the primary health services for NCDs? Please explain why.
   - Is the any community involvement in the management of NCDs, if yes please explain.
   - Are there any promotion/education campaigns/activities being conducted on NCDs?
2. To what extent do you see the budget impacting primary health services for NCDs? Please elaborate *[This question is for DHOs].*
   - Do you have fixed funds allocated for NCD programs?
   - Do you have organizations funding for NCD focused programs?
   - What is the annual budget for health facility with respect to NCDs? Has the budget increased over the last three years?
3. How do you describe the health workforce for NCDs at this facility in terms of size and capacity? How does this affect NCD disease specific care? Please explain.
4. What medicines and drugs/technology are available for NCD diagnosis/treatment and prevention at this facility?
   - How do you procure these drugs/technology?
   - How long does the supply last on average?
   - How do they meet the needs of the target population? Please explain.
   - How do you perceive the quality of these services?
   - What is their accessibility? What factors limit accessibility of these services?

Probe: how about drugs and technologies for diabetes?

1. Please describe the health information system for NCDs in your facility. *[This question is for DHOs].*
   - What needs exist and how does this health information system meet those needs? Please explain.
   - How do you perceive the quality of health information data?
   - Do you have system that monitors patients’ adherence to treatment as well as recall system for dropouts?
2. What are the most vital needs of this facility that if provided would ensure an increase in the capacity of NCD management in your catchment area?
   - Probe: Leadership and governance, Service delivery, Financing, Information, Health workforce, Medical supplies and technology, information.
   - Probe: what are the most vital needs to provide diabetes care?
3. Is there anything you would like to add?

Thank you very much for the time you spent in answering my questions today.
