# Supplementary material for: Primary health care preparedness to integrate diabetes care in Blantyre, Malawi: A mixed methods study
Source: PLoS One. 2024 May 21;19(5):e0303030. doi: 10.1371/journal.pone.0303030 (PMC11108178; doi:10.1371/journal.pone.0303030)
Supplement: Attachment — Submitted filename: PONE-D-23-35256_review.pdf [file pone.0303030.s003.pdf]

PLOS ONE

Primary healthcare preparedness to integrate diabetes care in Blantyre, Malawi: a mixed methods study

--Manuscript Draft--

**Manuscript Number:**	PONE-D-23-35256
**Article Type:**	Research Article
**Full Title:**	Primary healthcare preparedness to integrate diabetes care in Blantyre, Malawi: a mixed methods study
**Short Title:**	Primary healthcare preparedness to integrate diabetes care in Blantyre, Malawi
**Corresponding Author:**	Chimwemwe Kwanjo Banda<br>Malawi-Liverpool-Wellcome Clinical Research Programme<br>Blantyre, Blantyre MALAWI
**Keywords:**	healthcare system; non-communicable disease; self-management support; needs assessment; Health financing
**Abstract:**	Background: There is limited access to diabetes care services at primary care facilities in Malawi. Assessing the capacity of facilities to provide diabetes care is an initial step to integrating services at primary care.<br>Aim: To assess the preparedness for delivering diabetes services at primary care level within the Blantyre District Health Office (DHO) to support the response to NCD epidemic in Malawi.<br>Setting: Blantyre DHO primary care facilities.<br>Materials and methods: A mixed methods approach nested in a national needs assessment for NCD response in Malawi was used. Fourteen primary healthcare facilities from Blantyre DHO were assessed. A tool adapted from the WHO rapid assessment questionnaire was used to identify human resource, equipment, supplies, and medication needed for comprehensive diabetes care. Descriptive statistics were done to analyze the quantitative data. Fisher's exact test was used to assess if there was a statistically significant difference between urban and rural facilities. Seventeen health care workers from the selected facilities participated in key informant interviews. Framework analysis method guided the qualitative data analysis. The quantitative and qualitative data were merged and displayed jointly.<br>Results: The quantitative assessment showed that none of the facilities assessed had capacity to provide all the interventions recommended by WHO for diabetes care at primary level. Eight (57%) of the facilities had the capacity to diagnose diabetes, monitor glucose, prevent limb amputations and manage hypoglycemia and hyperglycemia. Four themes emerged from the qualitative data: differences in level of preparedness and implementation of diabetes care; disparities in resources between urban and rural facilities; low utilization of diabetes services; and strategy and policy recommendations for improvement of diabetes care.<br>Conclusion: Inadequate health financing resulted in significant disparities in the available resources between the rural and urban facilities to offer diabetes care services. There is need to develop national policies and guidelines for diabetes care to strengthen the capacity of primary care facilities to facilitate achievement of universal health coverage.
**Order of Authors:**	Chimwemwe Kwanjo Banda
	Ndaziona Peter Kwanjo Banda
	Belinda Thandizo Gombachika
	Moffat Joha Nyirenda
	Mina Hosseinpour
	Adamson Sinjani Muula
**Additional Information:**	
**Question**	**Response**

Powered by Editorial Manager® and ProduXion Manager® from Aries Systems Corporation

**Financial Disclosure**<br><br>Enter a financial disclosure statement that describes the sources of funding for the work included in this submission. Review the submission guidelines for detailed requirements. View published research articles from *PLOS ONE* for specific examples.<br><br>This statement is required for submission and **will appear in the published article** if the submission is accepted. Please make sure it is accurate.<br><br>**Funded studies**<br>Enter a statement with the following details:<br>• Initials of the authors who received each award<br>• Grant numbers awarded to each author<br>• The full name of each funder<br>• URL of each funder website<br>• Did the sponsors or funders play any role in the study design, data collection and analysis, decision to publish, or preparation of the manuscript?<br><br>Did you receive funding for this work?	Yes
Please add funding details.<br>as follow-up to "**Financial Disclosure**<br><br>Enter a financial disclosure statement that describes the sources of funding for the work included in this submission. Review the submission guidelines for detailed requirements. View published research articles from *PLOS ONE* for specific examples.<br><br>This statement is required for submission and **will appear in the published article** if the submission is accepted. Please make sure it is accurate.	Research reported in this publication was supported by the National Heart, Lung, And Blood Institute of the National Institutes of Health under Award Number U24HL136791. The content is solely the responsibility of the authors and does not necessarily represent the official views of the National Institutes of Health.<br>This research was also supported by the Consortium for Advanced Research Training in Africa (CARTA). CARTA is jointly led by the African Population and Health Research Center and the University of the Witwatersrand and funded by the Carnegie Corporation of New York (Grant No--B 8606.R02), Sida (Grant No:54100029), the DELTAS Africa Initiative (Grant No: 107768/Z/15/Z). The DELTAS Africa Initiative is an independent funding scheme of the African Academy of Sciences (AAS)'s Alliance for Accelerating Excellence in Science in Africa (AESA) and supported by the New Partnership for Africa's Development Planning and Coordinating Agency (NEPAD Agency) with funding from the Wellcome Trust (UK) (Grant No: 107768/Z/15/Z) and the UK government, the statements made and views expressed are solely the responsibility of the fellow.

**Funded studies**<br>Enter a statement with the following details:<br>• Initials of the authors who received each award<br>• Grant numbers awarded to each author<br>• The full name of each funder<br>• URL of each funder website<br>• Did the sponsors or funders play any role in the study design, data collection and analysis, decision to publish, or preparation of the manuscript?<br><br>Did you receive funding for this work?"	
Please select the country of your main research funder (please select carefully as in some cases this is used in fee calculation).<br>as follow-up to "**Financial Disclosure**<br><br>Enter a financial disclosure statement that describes the sources of funding for the work included in this submission. Review the submission guidelines for detailed requirements. View published research articles from *PLOS ONE* for specific examples.<br><br>This statement is required for submission and **will appear in the published article** if the submission is accepted. Please make sure it is accurate.<br><br>**Funded studies**<br>Enter a statement with the following details:<br>• Initials of the authors who received each award<br>• Grant numbers awarded to each author<br>• The full name of each funder<br>• URL of each funder website<br>• Did the sponsors or funders play any role in the study design, data collection and analysis, decision to publish, or preparation of the manuscript?<br><br>Did you receive funding for this work?"	KENYA - KE
**Competing Interests**	The authors have declared that no competing interests exist.

Powered by Editorial Manager® and ProduXion Manager® from Aries Systems Corporation

Use the instructions below to enter a competing interest statement for this submission. On behalf of all authors, disclose any competing interests that could be perceived to bias this work—acknowledging all financial support and any other relevant financial or non-financial competing interests.<br><br>This statement is required for submission and **will appear in the published article** if the submission is accepted. Please make sure it is accurate and that any funding sources listed in your Funding Information later in the submission form are also declared in your Financial Disclosure statement.<br><br>View published research articles from *PLOS ONE* for specific examples.<br><br>**NO authors have competing interests**<br><br>Enter: *The authors have declared that no competing interests exist.*<br><br>**Authors with competing interests**<br><br>Enter competing interest details beginning with this statement:<br><br>*I have read the journal's policy and the authors of this manuscript have the following competing interests: [insert competing interests here]*<br><br>* typeset	
**Ethics Statement**<br><br>Enter an ethics statement for this submission. This statement is required if the study involved:<br><br>• Human participants<br>• Human specimens or tissue<br>• Vertebrate animals or cephalopods<br>• Vertebrate embryos or tissues	Ethical clearance to conduct the study was granted by the College of Medicine Research Ethics Committee (COMREC) (Reference: P.07/19/2743). Permission to access the study sites was further sought from the Blantyre District Health Officer. Participation in the study was voluntary, and participants for the key informant interviews had to sign an informed consent, which explained their rights before taking part in the interview. All identifiable data of participants were removed from the transcripts, and consent forms were stored in lockable cabinets where access was limited to the principal investigator to maintain participants’ confidentiality.

Powered by Editorial Manager® and ProduXion Manager® from Aries Systems Corporation

• Field research<br><br>Write "N/A" if the submission does not require an ethics statement.<br><br>General guidance is provided below. Consult the submission guidelines for detailed instructions. **Make sure that all information entered here is included in the Methods section of the manuscript.**	

Powered by Editorial Manager® and ProduXion Manager® from Aries Systems Corporation

**Format for specific study types**<br><br>**Human Subject Research (involving human participants and/or tissue)**<br>• Give the name of the institutional review board or ethics committee that approved the study<br>• Include the approval number and/or a statement indicating approval of this research<br>• Indicate the form of consent obtained (written/oral) or the reason that consent was not obtained (e.g. the data were analyzed anonymously)<br><br>**Animal Research (involving vertebrate animals, embryos or tissues)**<br>• Provide the name of the Institutional Animal Care and Use Committee (IACUC) or other relevant ethics board that reviewed the study protocol, and indicate whether they approved this research or granted a formal waiver of ethical approval<br>• Include an approval number if one was obtained<br>• If the study involved *non-human primates*, add *additional details* about animal welfare and steps taken to ameliorate suffering<br>• If anesthesia, euthanasia, or any kind of animal sacrifice is part of the study, include briefly which substances and/or methods were applied<br><br>**Field Research**<br><br>Include the following details if this study involves the collection of plant, animal, or other materials from a natural setting:<br>• Field permit number<br>• Name of the institution or relevant body that granted permission	
**Data Availability**<br><br>Authors are required to make all data underlying the findings described fully available, without restriction, and from the time of publication. PLOS allows rare exceptions to address legal and ethical concerns. See the PLOS Data Policy and FAQ for detailed information.	Yes - all data are fully available without restriction

A Data Availability Statement describing where the data can be found is required at submission. Your answers to this question constitute the Data Availability Statement and **will be published in the article**, if accepted.<br><br>**Important:** Stating 'data available on request from the author' is not sufficient. If your data are only available upon request, select 'No' for the first question and explain your exceptional situation in the text box.<br><br>Do the authors confirm that all data underlying the findings described in their manuscript are fully available without restriction?	
**Describe where the data may be found in full sentences. If you are copying our sample text, replace any instances of XXX with the appropriate details.**<br><br>• If the data are **held or will be held in a public repository**, include URLs, accession numbers or DOIs. If this information will only be available after acceptance, indicate this by ticking the box below. For example: *All XXX files are available from the XXX database (accession number(s) XXX, XXX.*).<br>• If the data are all contained **within the manuscript and/or Supporting Information files**, enter the following: *All relevant data are within the manuscript and its Supporting Information files.*<br>• If neither of these applies but you are able to provide **details of access elsewhere**, with or without limitations, please do so. For example:<br><br>*Data cannot be shared publicly because of [XXX]. Data are available from the XXX Institutional Data Access / Ethics Committee (contact via XXX) for researchers who meet the criteria for access to confidential data.*<br><br>*The data underlying the results presented in the study are available from (include the name of the third party*	The datasets used and/or analysed during the current study are available from https://figshare.com/articles/dataset/NCD_focused_implimentation_research_needs_assessment_NCD_BRITE_xlsx/14540391

and contact information or URL).<br>• This text is appropriate if the data are owned by a third party and authors do not have permission to share the data.<br><br>* typeset	
Additional data availability information:	

Manuscript
Click here to access/download;Manuscript;Banda et al 2023 Manuscript.docx

Primary health care preparedness to integrate diabetes care in Blantyre, Malawi: a mixed methods study

Authors: Banda, C. K[1,2*], Ndaziona Peter Kwanjo Banda[3], Gombachika, B.T[4], Nyirenda, M.J[1,5,6], Hosseinpour M[6,7], Muula, A.S[1,6,8]

[1] School of Global and Public Health, Kamuzu University of Health Sciences, Blantyre, Malawi

[2]Public Health Group, Malawi Liverpool Wellcome Program, Blantyre, Malawi

[3]Department of Medicine, Kamuzu University of Health Sciences, Blantyre, Malawi

[4]School of Nursing, Kamuzu University of Health Sciences, Blantyre, Malawi

[5]Uganda MRC/UVRI Research Unit, Entebbe, Uganda

[6]NCD-BRITE Consortium, Kamuzu University of Health Sciences, Blantyre, Malawi

[7]University of North Carolina at Chapel Hill

[8]Africa Center of Excellence in Public Health and Herbal Medicine, Kamuzu University of Health Sciences, Blantyre, Malawi

***Corresponding author**

Email: cbanda@cartafrica.org **Cell:** +265884711313

Abstract

Background: There is limited access to diabetes care services at primary care facilities in Malawi. Assessing the capacity of facilities to provide diabetes care is an initial step to integrating services at primary care.

Aim: To assess the preparedness for delivering diabetes services at primary care level within the Blantyre District Health Office (DHO) to support the response to NCD epidemic in Malawi.

Setting: Blantyre DHO primary care facilities.

Materials and methods: A mixed methods approach nested in a national needs assessment for NCD response in Malawi was used. Fourteen primary healthcare facilities from Blantyre DHO were assessed. A tool adapted from the WHO rapid assessment questionnaire was used to identify human resource, equipment, supplies, and medication needed for comprehensive

diabetes care. Descriptive statistics were done to analyze the quantitative data. Fisher's exact test was used to assess if there was a statistically significant difference between urban and rural facilities. Seventeen health care workers from the selected facilities participated in key informant interviews. Framework analysis method guided the qualitative data analysis. The quantitative and qualitative data were merged and displayed jointly.

Results: The quantitative assessment showed that none of the facilities assessed had capacity to provide all the interventions recommended by WHO for diabetes care at primary level. Eight (57%) of the facilities had the capacity to diagnose diabetes, monitor glucose, prevent limb amputations and manage hypoglycemia and hyperglycemia. Four themes emerged from the qualitative data: differences in level of preparedness and implementation of diabetes care; disparities in resources between urban and rural facilities; low utilization of diabetes services; and strategy and policy recommendations for improvement of diabetes care.

Conclusion: Inadequate health financing resulted in significant disparities in the available resources between the rural and urban facilities to offer diabetes care services. There is need to develop national policies and guidelines for diabetes care to strengthen the capacity of primary care facilities to facilitate achievement of universal health coverage.

Key words: Healthcare system, non-communicable disease, self-management support, needs assessment, Health financing

Introduction

According to the Sustainable Development Goals (SDGs), countries are required to reduce the morbidity and mortality rates of four main non-communicable diseases (NCDs), including cardiovascular diseases (CVDs), diabetes mellitus (DM), chronic respiratory diseases (CRDs), and cancer (CA), by one-third in 2030 from the 2015 level [1]. The problem is expected to be more complex in resource limited low- and middle-income countries (LMICs), where health systems are faced with a dual burden of infectious diseases and rise in non-communicable chronic conditions[2]. With inadequate and uneven distribution of specialized care facilities, most patients with diabetes in LMICs depend on an already overburdened primary care for their health care needs [3]. Furthermore, the traditional orientation of health systems towards infectious disease

management, limited resources and fragmented primary care, pose an uphill task for the primary care physicians in managing patients with diabetes and comorbidities.

Strengthening and orienting the healthcare system to address the prevention and control of Non-Communicable Diseases (NCDs) NCD and the underlying social determinants is one of the priority areas for achieving universal health coverage in Malawi [4]. In Malawi, diabetes mellitus is one of the NCDs that cause significant morbidity and mortality, yet access to preventive care and treatment is a challenge for many [5]. The primary healthcare system has a vital role in prevention and control of diabetes and other NCDs [6,7].

Malawi adopted the World Health Organization (WHO) framework for strengthening equity and efficiency of primary healthcare for NCDs in low and middle-income countries (LMICs) and launched a national action plan for NCD prevention and management in 2013 [8]. However, availability of diabetes services at primary care facilities especially in rural areas continue to be inconsistent in the country[8–10]. In Blantyre district, many people living with diabetes attend the diabetes clinic at Queen Elizabeth Central hospital (QECH), a specialized public referral and teaching hospital in the southern region of Malawi [11]. This is despite the existence of primary care facilities managed by the Blantyre district health office (DHO) within the district. Centralizing diabetes services limits accessibility and compromises care due to large patient numbers against staff shortages, limited laboratory capacity, and diabetes medication stock outs [12]. In our previous study, we found that self-management support for people with diabetes attending the QECH diabetes clinic was inadequate due to staff shortages and high client turnover at the facility [13]. These findings highlighted the need of increasing access to diabetes services through primary care health facilities.

The lack of baseline data on burden of diabetes and the capacity of facilities to provide diabetes services is one of the challenges to integration of diabetes services into primary care services in many LMICs like Malawi [12,14]. Studies assessing the capacity of health systems to deliver diabetes care in other sub-Saharan countries (Ghana [15], Zambia [16], Tanzania [17], Democratic Republic of Congo [16]) revealed that lack of equipment and basic medicines was common, however, each country had its own strengths and specific needs. Therefore, locally generated research data was thus needed to guide policy makers and service providers in response to NCDs and to identify existing resource and service in managing diabetes at primary care level in Malawi

[12]. Introducing diabetes service at the primary care level without understanding the burden of disease and the capacity of the facilities to render such services may be cost ineffective and a waste of resource. Conducting a needs assessments before integrating diabetes services into primary care is therefore essential in addressing the knowledge-practice gap that exists [15,18,19].

The purpose of the assessment was to determine the preparedness of the primary care facilities in Blantyre DHO to provide diabetes services to support the response to the NCD epidemic in the Malawian health sector. We sought to determine the burden of diabetes; the size and capacity of human resource to manage diabetes; and availability of basic equipment, medicine and guidelines for the diagnosis and treatment of diabetes. The study was nested in a larger study conducting a national needs assessment for NCD response in Malawi [20].

Materials and methods

Study design

We conducted a mixed methods study (Fig 1) to understand the context through which primary care services are provided in Blantyre DHO primary care facilities. A concurrent triangulation mixed methods strategy was used to collect the data where by qualitative and quantitative data were collected at the same time then analyzed [21]. The mixed methods approach was used to maximize the strengths and to minimize the weaknesses inherent in each approach [21].

Fig 1. Study design

Study context

The study setting was Blantyre DHO primary care facilities. In Malawi primary care services are provided through health centers. The health centers provide outpatient and maternity services. Blantyre DHO has 28 health facilities of which 18 are health centers the other 10 are community dispensaries or health posts. Six of the health centers are located in urban area and twelve are rural. Unlike other DHOs in Malawi, the Blantyre DHO has no secondary level facility. All clients needing further treatment at the Blantyre DHO primary care facilities are referred to QECH, a regional tertiary hospital. Staffing at primary care facilities mostly

comprise non-physician providers (medical assistants, clinical technicians or clinical officers) and nursing and midwifery technicians or registered nurses [22,23].

Fourteen primary care facilities (seven urban and seven rural), from Blantyre DHO were included in the assessment. These facilities included six health centers and one dispensary (offering primary care services) from Blantyre urban, and seven rural health centers randomly selected and equaled with the number of the urban facilities. The fourteen facilities were considered enough to represent all the primary care facilities in assessing the capacity of Blantyre DHO to provide diabetes care services.

Sampling

Quantitative

Fourteen facilities (seven urban and seven rural) from Blantyre DHO were included in the assessment. At the time of the assessment, Blantyre DHO had a total of 19 health centers (seven urban and 12 rural). All the urban health center, and seven rural health centers randomly selected were included in the assessment. To randomly select the rural health centers, names of all the facilities were listed in an alphabetical order and numbered from one to twelve in Microsoft Excel. The RANDBETWEEN function in Microsoft excel was used to select the seven rural facilities to be assessed. The fourteen facilities were considered enough to represent all the primary care facilities in assessing the capacity of Blantyre DHO to provide diabetes care services.

Qualitative

One or two healthcare workers were purposively selected from each participating health center to participate in key informant interviews. The participating healthcare workers included medical doctors, clinical officers, clinical technicians, medical assistants, nurses, pharmacy and physiotherapy departments' personnel. These people were recruited because of their knowledge and experience of working with people with diabetes or other NCDs at Primary care level for at least six months.

Ethical consideration

Ethical clearance to conduct the study was granted by the College of Medicine Research Ethics Committee (COMREC) (Reference: P.07/19/2743). Permission to access the study sites was

further sought from the Blantyre District Health Officer. Participation in the study was voluntary, and participants for the key informant interviews had to give a written consent. All identifiable data of participants were removed from the transcripts, and consent forms were stored in lockable cabinets where access was limited to the principal investigator to maintain participants' confidentiality.

Data collection

All data collection was from 19th December 2019 and to 31st March 2020.

Quantitative study

Quantitative data were collected using a needs assessment tool (Appendix 1) which was developed to help assess the human and infrastructural capacity needs for NCD Response in health facilities in Malawi. The tool adapted the WHO questionnaire for the assessment of capacity to prevent and manage major NCDs in primary care centers in low-resource settings [24]. The tool has different sections to capture the following details: health facility profile; human resource and skills profile; facility-based diabetes prevalence; clinics and services; equipment; medicines and sundries; laboratory; costs related to diabetes; and referral system. For this present study, the assessment focused on diabetes services. Under each section, some items were added or removed from the original tool to make it comprehensive and context specific for the Malawi healthcare service.

The researcher or the research assistants administered the data tool to facility in-charges or their deputies and pharmacy personnel. Information on staff records for all facilities was collected from the Blantyre DHO human resource officer. Disease prevalence data for all facilities was obtained from the Blantyre DHO health management information system (HMIS) officers.

Qualitative data

Sixteen key informant interviews were conducted with healthcare worker working at primary care facilities within Blantyre DHO. The first author or a research assistant, made appointments for interviews with the person in charge of the facilities or their representatives, in person or through a phone call. An information sheet was given to those who met the eligibility criteria (aged 18 and above, employed by ministry of health and having worked at the facility for at least six months) for them to read about the study. If they accepted to be interviewed, they were requested to sign the consent form. The research team member who obtained the consent also signed to confirm the person was informed about the study and was given an information sheet to

read. The interviews were conducted on the day convenient to the participant in English as all the participants that were recruited were fluent in the language. A structured interview guide (Appendix 2) adapted from the Malawi national needs assessment study was used for the key informant interviews [20]. To ensure alignment of the needs assessment with governments' health agenda, the sections of the interview guide were according to the following priority areas of the Malawi National Health plan: service delivery; preventive health and social determinants of health; leadership and governance; health financing; human resource for health; medicines, medical supplies, medical equipment and infrastructure; and health information and research [4]. All interviews were recorded using a voice recorder and short notes were jotted during the interviews.

Data analysis

Quantitative data

For the quantitative data, completed checklists and records were entered into a database created in Microsoft Excel. The data were exported into Stata Software version 14 for analysis. Descriptive statistics were calculated and presented as frequencies and percentages. Using the WHO guidelines for prevention and control of NCDs at primary care facilities in low resource settings, we considered individual facilities' capacity to provide diabetes services in six areas: (1) diabetes diagnosis; (2) glucose control; (3) reducing the risk of cardiovascular disease and diabetic nephropathy; (4) prevention of lower limb amputations; (5) prevention of blindness; and (6) management of severe hypoglycaemia and hyperglycaemic emergencies. Facilities were considered prepared if they had a) staff trained in diabetes management (during pre-service or in-service), b) necessary equipment and c) medications. Facilities were thus classified as prepared if they had all the three factors or not prepared if they had any of those factors missing. The differences in preparedness between urban and rural facilities was tested using the Fisher's exact test of association. A significance level of alpha equal to 0.05 was used to determine the statistical significance.

Qualitative data

Qualitative interview audios were transcribed verbatim by the first author, and where applicable they were translated from the vernacular language to English. Copies of transcripts were emailed to participants for them to comment if the transcript truly represents their views (member checking) [25]. The framework analysis method, according to Ritchie and Lewis guided the data

analysis [26]. The first and second author read the transcripts to familiarize themselves with the data, then coded the data and agreed on the initial analytical framework. The initial analytical framework was based on the Malawi National Health Plan priority areas, and the interview guide and was applied to all the data. The coded data were then charted into corresponding cell of the framework. The analytical framework was further refined in an iterative process as new themes emerged from the data. The final thematic framework had four main themes.

Results

Qualitative findings and quantitative results were integrated by merging to support each other [21]. The merged data are jointly displayed [27] in tables to show areas of convergence based on the qualitative themes. Four major themes emerged from the qualitative data: differences in level of preparedness and implementation of diabetes care; lack of resources; low utilization of diabetes services; and strategy and policy recommendations for improvement of diabetes care.

Table 1 shows the characteristics of participants in the qualitative interviews with staff from the 14 facilities that were included in the assessment.

Table 1: Qualitative interviews participants' characteristics

**Variable**	**Characteristic**	**n = 16**
**Sex**	Male	5
	Female	11
**Age**	20 – 29 years	10
	30 – 39 years	6
**Occupation**	Medical doctor	6
	Clinical Officer/ technician	3
	Medical Assistant	5
	Registered nurse/midwife	1
	Rehabilitation technician	1
**Work experience**	1 – 5years	11

	6 – 10 years	4
	11 – 15 years	1

Theme 1: Differences in capacity and implementation of diabetes care

Six of the fourteen facilities that were assessed had a diabetes clinic. The actual services provided and capacity to offer recommended interventions differed among the facilities. None of the facilities had the capacity to implement all the WHO recommended interventions for diabetes care at primary care facilities (Table 2).

Table 2: Facilities with capacity or offering WHO recommended intervention for diabetes at primary level and source of guidelines

**Intervention**	**Facilities offering service (N= 14)**	**Facilities with capacity (N= 14)**	**Illustrative quotes**
Diabetes diagnosis	9 (64%)	9 (64%)	*"We cannot diagnose diabetes. We don't have a glucometer."* Participant **12/medical assistant/male.**<br><br>*"For diagnosis, we are only able to really diagnose clinically… diabetes with the glucosticks* and *we have glucometers. For treatment, for diabetes we only have metformin and glibenclamide that we are able to give".* P**articipant7/medical doctor/ female.**<br><br>*"We have clinics in our district but most of our clinics [only] do drug refill."* **Participant1/medical doctor/female.**
Glucose control	6 (43%)	8 (57%)	
Reducing the risk of cardiovascular disease and diabetic nephropathy	3 (21%)	3 (21%)	
Prevention of lower limb amputations	6 (43%)	8 (57%)	
Prevention of blindness	2 (14%)	2 (14%)	
Management of severe hypoglycaemia and	9 (64%)	9 (64%)	

hyperglycaemic emergencies			*"If we could have been doing the kidney function test. We check the urea, the creatinine, if our lab had those machines, at least we could decrease the burden at Queens."* ***Participant4/medical doctor/male.*** <br>*"If we could have the ability to have frequent blood checks . . .The other tests like the biochemistry tests, that would also be helpful . . ."* ***Participant7/medical doctor/female***
All interventions	0 (0%)	0 (0%)	

Theme 2: Lack of resources

Lack of adequate and appropriately trained human resources, medicines, infrastructure and diagnosis equipment was identified as a main problem at all facilities.

Human resources

Human resource needs were cited as one of the most vital needs to improve care. Fifty two percent of the human resource available at the assessed facilities were Health Surveillance Assistants (HSAs). Other staff categories that were more common were nurse midwife technicians (24.15%) then medical assistants (10.1%). Only four (0.6%) of all the available staff reported to have attended a one-time training in NCD management. The key informants perceived that there was need for training and mentorship to equip those that lacked knowledge and skills in diabetes management.

"We need the staff to be trained so they can be updated. Things are changing and we were trained way back." ***Participant8/medical assistant/male.***

"Some of those cadres, they are not really trained, or they don't have the best experience when it comes to managing special cases or when say hypertension has complicated to heart failure. They are not the best people to actually manage those conditions. So we have no choice, but to still have them on the rota because we don't have too many people. . ." ***Partcipant7/medical doctor/female.***

It was also reported that some of the practitioners lacked confidence to handle diabetes

"In other facilities the services are there but the clinicians are afraid to take it up, I don't know why… so mostly they would just refer the patient to Queens or tell them to go to another health center where there is [diabetes] clinic". ***Participant2/medical doctor/female***

All facilities cited shorted of staff as a major challenge and the rural facilities were significantly more under-staffed compared to the urban facilities (Table 2).

Table 2: Number of different categories of human resources per facility

Staff category	Urban Median (IQR)	Rural Median (IQR)	P value	Illustrative quote
Health Surveillance assistants	37 (20 – 42)	14 (8 – 20)	0.0072	*"We have a shortage of staff. For example, for us clinician, we are just two of us. That's a big challenge. Even the other cadres, like the HSAs are not enough. The main challenge is staffing. Like today, my colleague lost a relation and has gone out for a funeral. So I am the only one covering the facility for day and night shift."* ***Participant13/medical assistant/female.*** <br>*"I think the [human resource] size is small, very small against the patients that we receive. . . we have just one clinician who is doing all the screening, it affects the care that he is going to give because the same person is supposed to check the blood pressure, the same person is supposed to do the weighing scale and everything, so it's a*
Nurse midwife technicians	15 (14 – 18)	4 (3 – 11)	0.0120	
Medical Assistants	5 (4 – 7)	2 (1 – 3)	0.0067	
Community midwives	1 (1 – 3)	0 (0 – 1)	0.0451	
Registered nurse midwives	4 (3 – 4)	0 (0 – 3)	0.0038	
Clinical technicians	2 (2 – 6)	0 (0 – 2)	0.0091	
Laboratory technician	1 (1 – 2)	0 (0 – 0)	0.0095	
Medical doctor	1 (1 – 1)	0 (0 – 0)	0.0005	
Clinical officers	1 (0 – 2)	0 (0 – 0)	0.0245	
Environmental Health	0 (0 – 1)	0 (0 – 0)	0.0245	

Laboratory assistant	1 (0 – 1)	0 (0 – 0)	0.0241	*little bit tiresome, and for example if I have fifteen or twenty patients, the way you are going to see those patients is very different than if you have a little more hands in.*
Rehabilitation Assistant	0 (0 – 0)	0 (0 – 0)	0.9165	*" **Participant5/clinical officer/ female"***

Medicines

The diabetes medications stocked at the facilities were biguanide (Metformin) and sulphonylurea (Glibenclamide). Metformin was available at eight (57.14%) of the facilities, of which seven were urban and one was rural. All the facilities reported experiencing metformin stock out in the last quarter while five reported metformin stock out in the past year. Glibenclamide was available at seven (50%) of the facilities of which six were urban and one was rural. Two facilities reported stock out of glibenclamide in the last quarter and four facilities in the past year. Reasons for not stocking oral hypoglycemic medications were that the drugs are not supplied when ordered, or the clinicians were not confident to prescribe the drugs, or because the facility does not attend to many people with diabetes. All (100%) the facilities did not have any kind of insulin. Table 3 below shows the number of urban and rural facilities that stocked the different classes of diabetes medication.

Table 3: Stocking of diabetes medication between urban and rural facilities

Drug category	Urban N = 7	Rural N = 7	P value	Illustrative quote
Biguanide	7 (100%)	1 (14%)	0.002	*"We don't have diabetes drugs. Because we don't order. We mostly don't know how to manage such cases."* **participant10/medical assistant/female.**
Sulfonylurea	6 (86%)	1 (14%)	0.015	
Insulin	0 (0%)	0 (0%)		*"But the actual medicine like metformin or glibenclamide, we actually don't have… We don't stock that here. I'd say I haven't seen it in stock since I have*

				been here. . .They don't supply. ***Participant11/clinical technician/male.*** *"Of course they don't give us insulin, insulin is given at Queens."* ***Participant2/medical doctor/female.***

Antihypertensives were available at most facilities. Hydrochlorothiazide was available at 13 (92.86%) facilities. Calcium channel blockers like nifedipine at 9 (64.29%) of the facilities. Beta blockers like propranolol were available at 6 (42.86 %) of the facilities. ACE inhibitors were available at only 3 (21.43%) of the facilities. The antibiotics mostly available at the facilities were benzathine (92.86%), ceftriaxone (85.71%) and amoxillin (57.14%). For anticoagulants, warfarin and protamine were not available at any of the facilities while one (7.14%) facility had heparin. Other medications like statins were available at only one (7.14%) facility. Only five (35.71%) facilities reported that they receive the types of the medication they ask for, and two (14.29%) receive the quantities they order. Six (42.86 %) had no refrigerator or air con in the pharmacy

Infrastructure and Equipment

None of the facilities had a specific room for diabetes care. At facilities which had diabetes service, the clinics were conducted at the out-patient department. A number of facilities did not have any of the essential basic equipment (Table 4).

Table 3: Availability of essential basic equipment in urban and rural facilities

Item	Urban Median (IQR)	Rural Median (IQR)	P value	Illustrative quote

Glucometers	1 (1 – 1)	0 (0 – 1)	0.0072	*"Yes, we have the glucometer but the sticks"* ***participant1/medical doctor/female.***<br><br>*"Another thing is to do with the BP cuffs, most times we run out of batteries. Sometimes we use a manual machine, but if using the manual one and you have so many patients, it takes time. It's better using the digital one ... We are able to check for RBS but most times we run out of stock of the sticks, so it usually difficult to monitor."****Participant11/medical assistant/male.***<br>*"We have BP cuff, however for most times, we have problems with batteries. So sometimes we use our own money to buy the batteries so that we can work. Other times the patients themselves when we tell them that we don't* have batteries,
Mercury BP†	0 (0 – 1)	0 (0 – 0)	0.1422	
Aneroid BP	1 (0 – 2)	0 (0 – 1)	0.1967	
Automatic BP	2 (1 – 4)	1 (0 – 2)	0.0654	
Standard BP	2 (1 – 6)	3 (2 – 4)	0.6954	
Alternate cuff	0 (0 – 1)	0 (0 – 0)	0.4760	
Bathroom scale	1 (0 – 2)	0 (0 – 1)	0.0797	
Hospital scale	1 (1 – 3)	1 (1 – 3)	0.7362	
Height meter	0 (0 – 3)	1 (0 – 1)	0.7333	
Ophthalmoscope	0 (0 – 0)	0 (0 – 0)	0.3173	
Snellen's chart	0 (0 – 0)	0 (0 – 0)	0.3173	
BMI‡ Chart	1 (0 – 1)	0 (0 – 1)	0.4751	

Monofilament	0 (0 – 1)	0		some *will say "but I want my BP to be checked, so I will go to buy the batteries". So that's the challenge we have with hypertension. While with diabetes, we don't have any equipment"* ***Participaant13/medical assistant/female***

† Blood Pressure;   ǂ Body Mass Index

Theme 3: Low utilization of diabetes services

In facilities where diabetes services were available, utilization was low. Participants perceived that utilization was low due to lack of community awareness on the existence of the services and lack of trust in the services.

None of the facilities had community awareness campaigns, outreach or education programs for diabetes. The median (IQR) number of clients seen per facility due to diabetes in the previous twelve months was 174 (151 – 233) in the urban and 97 (42 – 110) in the rural (p = 0.0017). Key informants from urban facilities were of the view that many of the clients with diabetes were not utilizing the primary care facilities despite increase in diabetes cases within the catchment areas.

> *"We don't see many cases of diabetes... most of them go to Queens [QECH]. . . ."* **Participant10/medical assistant/female.**

> *"they are used to Queens {QECH] which is our main tertiary facility and referral facility, so these other clinics are not well patronized because our community outside there don't know that we have those services…"* **Participant1/medical doctor/female.**

inconsistencies in availability of diabetes medicines and supplies at the primary care facilities was the other reason cited for low client turnover. One participant had this to say:

> *"We also have stock outs, that's often the main reason why people don't come. They say even if we go, we don't find the drugs."* **Participant6/medical doctor/male.**

Theme 4: Strategy and policy recommendations for improvement of diabetes care

The participants suggested some strategies and policy recommendations to improve the primary health services for diabetes. Some suggested having national programs for NCDs same way it is for communicable diseases.

> *"There should be national programs for NCD like it is with other diseases, for example end TB."* ***Participant8/medical assistant/male.***

Other participants suggested screening services for NCDs at OPDs.

> *"So I believe just to enhance on screening of NCDs, maybe if we would have rules established for basic things like vital signs for all the OPD patients so we can pick up patients that don't know.,. . .. So enhancing primary screening to all OPD patients can be helpful."* ***Partcipant3/clinical officer/female.***

Another suggestion was on provision for management guidelines. The Malawi Standard Treatment Guidelines (MSTG) and the QECH medical department clinical book (referred to as the blue book) were available in 10 (71.43%) of the facilities. These guidelines were available as hard copies or electronic copies.

> *"I think if we could have support with the guidelines. As I said most of the recommended guidelines that I know personally are the blue book, which is not really distributed to us."* ***Participant7/medical doctor/female***

> *"We have soft copies. Which we share on our phones . . . the one we receive is from Queens. We get them from Queens."* ***Participant5/medical doctor/female***

These guidelines were not accessible to all service providers as one participant had this to say:

> *"As for me, I have never seen any guidelines."* ***Participant13/medical assistant/female***

The existing guidelines were however perceived as not offering adequate guidance to implementing diabetes services

"Not all [diabetes] patients need to be seen at Queens or to be seen by doctors. Currently I know that some facilities are able to give a few of the medicine but they don't really offer reviews or basic checkups for those [diabetes] patients. So if that could be standardized to make sure it's happening, that would be good". ***Participant7/medical doctor/female***

Discussion

The needs assessment suggested that primary care facilities within Blantyre DHO were not adequately prepared to integrate WHO guidelines for diabetes care [28]. Although some facilities were implementing some diabetes care, none of the facilities had the capacity to implement all interventions recommended by WHO (diabetes diagnosis; glucose control; reducing the risk of cardiovascular disease and diabetic nephropathy; prevention of lower limb amputations; prevention of blindness; and management of severe hypoglycaemia and hyperglycaemic emergencies). Only few of the facilities had capacity to implement interventions for reducing the risk of cardiovascular diseases, diabetic nephropathy or retinopathy. The urban facilities had capacity for implementing more interventions for diabetes than the rural facilities. Therefore, when implementing the WHO Package for Essential NCD (PEN) interventions [6], individual facility capacity should be taken into consideration to ensure appropriate allocation of resources.

The assessment revealed that lack of resources limited the capacity of primary care facilities to provide diabetes services. There were limitations in the size and capacity of human resources, lack of equipment and supplies, and shortage of diabetes medication for the primary care facilities to provide diabetes care. Although all the urban facilities had at least one medical doctor, shortage of some basic equipment like blood pressure machines, testing reagents for glucometers or urine dipsticks for urinalysis and shortage of first-line drug regimens for hypertension and diabetes made it difficult for them to provide optimal care. While in rural facilities, the majority of the staff were non-physicians (medical assistants or clinical technicians) [22] who might have had no pre-service or in-service training in diabetes management. This made it difficult for them to know what equipment or medications to stock for diabetes management, or how to assist people with diabetes. If offered appropriate training and support, non-physician practitioners can be a useful resource in management of diabetes at primary care level [29]. Our findings on the limited capacity for primary care facilities to

provide diabetes care in line with WHO recommendations concur with findings from other studies from within the region [15–17,30]. Some have however argued that the lack of preparedness to manage diabetes at primary care facility is a reflection of non-optimal healthcare systems in low income countries [31]. Low and inadequate health financing in the main reason for poor services at primary healthcare facilities and the reason many LMICs fail to achieve universal health coverage. [32]. Therefore, improving diabetes services at primary care level facilities will require improvement of all other services.

Another reason for inadequate diabetes services at primary care level is the lack of national policy and guidelines for management of diabetes [33]. Only 53% and 59% of African and low income countries respectively have an operational policy, strategy or action plan for diabetes [34]. Malawi is one of the counties without a diabetes policy, strategy or action plan. Although the national health policy has a statement on improving diabetes care in the country [4], the national health sector strategic plan II categorizes diabetes treatment as unaffordable at the primary level therefore it is excluded from the national basic health package [35]. The available national prescription guidelines were not comprehensive enough to guide prevention, diagnosis, and management of diabetes, or its complications [36]. Similarly, the other available handbook which the clinicians consulted focused more on acute care for diabetes as it was intended for use at a tertiary facility [37]. Therefore, the need for having national guidelines or policy diabetes management was expressed by most of the healthcare workers who took part in the interviews. With sound policies, financing and guidelines, primary care facilities in Malawi have successfully responded to infectious condition like malaria [38], tuberculosis[39] and Human Immunodeficiency Virus (HIV)[40] despite resource limitations in the health sector. Therefore the implication for policy is that to strengthen primary care facilities' capacity for diabetes care, there is need for national policy and guidelines for diabetes prevention and control.

Unfortunately, we also found that even where some diabetes care services were available, utilization of the services by surrounding communities was low. People with diabetes preferred to go to QECH, the regional referral hospital than their nearest primary care facilities. Local communities did not patronize the primary care facilities either due to lack of awareness on the existence of the services or due to lack of trust with in the primary care facilities to find the service they needed when they present there. Therefore, there is need to increase public

awareness on the available diabetes services at primary care level and ensuring consistency of available services. A study assessing the feasibility of decentralizing diabetes care at primary healthcare facilities in Zomba, Phalombe, Machinga and Mulanje districts in Malawi, managed to retain 73% of people with diabetes in care over a 12 months period [5]. Staff trainings in diabetes care, mentorship programs, drug supply system strengthening, record keeping and peer education are some of the strategies that were used in this study [5]. Similar strategies could be adopted in Blantyre DHO primary care facilities to strengthen the capacity of diabetes care and maximize reach to people requiring the services within the catchment area of the facilities. Additionally, equipment for diabetes diagnosis and monitoring of glucose control and diabetes complications should be consistently made available at the primary care facilities.

The strength of our study was that by utilizing a mixed methods approach to the assessment, we were able to include narrative explanations to support the figures, and each method compensated the other methods' weaknesses. Secondly the setting of our study allowed us to compare rural and urban facilities unlike other DHOs in the country which only have rural facilities. There were however some limitations to our study. Firstly, we are not able to make statistical generalizations from the findings as data were collected at primary care facilities from one DHO. Secondly, we relied on the informants reports, therefore there was a possibility of social desirability bias, where participants present themselves in the most favorable manner [41].

Conclusion

Integration of diabetes care at primary level facilitates the achievement of universal accesses to health care for all. However, shortage of skilled staff, medication and equipment limits the capacity of primary care facilities in Blantyre DHO to provide diabetes services. A national policy and guidelines for diabetes care are also needed to strengthen the capacity of primary care facilities. Strengthening health systems financing at primary care level is therefore needed to improve the services and close the existing knowledge-practice gap.

Acknowledgements

Research reported in this publication was supported by the National Heart, Lung, And Blood Institute of the National Institutes of Health under Award Number U24HL136791. The content is

solely the responsibility of the authors and does not necessarily represent the official views of the National Institutes of Health.

This research was also supported by the Consortium for Advanced Research Training in Africa (CARTA). CARTA is jointly led by the African Population and Health Research Center and the University of the Witwatersrand and funded by the Carnegie Corporation of New York (Grant No--B 8606.R02), Sida (Grant No:54100029), the DELTAS Africa Initiative (Grant No: 107768/Z/15/Z). The DELTAS Africa Initiative is an independent funding scheme of the African Academy of Sciences (AAS)'s Alliance for Accelerating Excellence in Science in Africa (AESA) and supported by the New Partnership for Africa's Development Planning and Coordinating Agency (NEPAD Agency) with funding from the Wellcome Trust (UK) (Grant No: 107768/Z/15/Z) and the UK government, the statements made and views expressed are solely the responsibility of the fellow.

Competing interest

None

References

1. ODDS. Transforming our world: the 2030 Agenda for Sustainable Development. United Nations New York, NY, USA 2015.

2. Dagenais GR, Gerstein HC, Zhang X, McQueen M, Lear S, Lopez-Jaramillo P, et al. Variations in diabetes prevalence in low-, middle-, and high-income countries: results from the prospective urban and rural epidemiological study. Diabetes Care 2016;39:780–7.

3. Kruk ME, Nigenda G, Knaul FM. Redesigning primary care to tackle the global epidemic of noncommunicable disease. Am J Public Health 2015;105:431–7.

4. Malawi Government. National Health Policy: Towards Universal Health Coverage. Lilongwe, Malawi: Ministry of Health; 2018.

5. Pfaff C, Malamula G, Kamowatimwa G, Theu J, Allain TJ, Amberbir A, et al. Decentralising diabetes care from hospitals to primary health care centres in Malawi.

Malawi Med J 2021;33:159–68.

6. World Health Organization. Package of Essential Noncommunicable disease (PEN) interventions for primary health care in low-resource settings. Geneva, Switzerland: World; 2010.

7. World Health Organization. Global action plan for the prevention and control of noncommunicable Diseases (2013 - 2020). Geneva, Switzerland: World Health Organization; 2013.

8. Ministry of Health. The Malawi Noncommunicable Diseases and Injuries Poverty Commission Report. 2018. doi:10.1002/da.22823.

9. Wood R, Viljoen V, Van Der Merwe L, Mash R. Quality of care for patients with non-communicable diseases in the Dedza District, Malawi. African J Prim Heal Care Fam Med 2015;7:1–8.

10. Assayed AA, Muula AS, Nyirenda MJ. The quality of care of diabetic patients in rural Malawi: a case of Mangochi district. Malawi Med J 2014;26:109–14.

11. Allain TJ, van Oosterhout JJ, Douglas GP, Joukes S, Gadabu OJ, Darts C, et al. Applying lessons learnt from the 'DOTS'tuberculosis model to monitoring and evaluating persons with diabetes mellitus in Blantyre, Malawi. Trop Med Int Heal 2011;16:1077–84.

12. Maher D, Sekajugo J, Harries AD, Grosskurth H. Research needs for an improved primary care response to chronic non- communicable diseases in Africa. Trop Med Int Heal 2010;15:176–81.

13. Banda CK, Gombachika BT, Nyirenda MJ, Muula AS. Self-management and its associated factors among people living with diabetes in Blantyre, Malawi: a cross-sectional study. AAS Open Res 2019;2:161.

14. Ali MK, Rabadán-Diehl C, Flanigan J, Blanchard C, Narayan KMV, Engelgau M. Systems and capacity to address noncommunicable diseases in low-and middle-income countries. Sci Transl Med 2013;5:181cm4-181cm4.

15. Nyarko KM, Ameme DK, Ocansey D, Commeh E, Markwei MT, Ohene S-A. Capacity

assessment of selected health care facilities for the pilot implementation of Package for Essential Non-communicable Diseases (PEN) intervention in Ghana. Pan Afr Med J 2016;25.

16. Mutale W, Bosomprah S, Shankalala P, Mweemba O, Chilengi R, Kapambwe S, et al. Assessing capacity and readiness to manage NCDs in primary care setting: gaps and opportunities based on adapted WHO PEN tool in Zambia. PLoS One 2018;13:e0200994.

17. Peck R, Mghamba J, Vanobberghen F, Kavishe B, Rugarabamu V, Smeeth L, et al. Preparedness of Tanzanian health facilities for outpatient primary care of hypertension and diabetes: a cross-sectional survey. Lancet Glob Heal 2014;2:e285–92.

18. Elias MA, Pati MK, Aivalli P, Srinath B, Munegowda C, Shroff ZC, et al. Preparedness for delivering non-communicable disease services in primary care: access to medicines for diabetes and hypertension in a district in south India. BMJ Glob Heal 2018;2:e000519.

19. Bawazir A, Al-Surimi K, Suwaidan SD, AlShehri AM, AlFarhan AI, Abolfotouh MA. Capacity and readiness of primary health care centers for implementation of the basic strategy for prevention and control of non-communicable diseases in Saudi Arabia. A case study from the Ministry of National Guard-Health Affairs, Riyadh, Saudi Arabia. Saudi Med J 2019;40:614.

20. van Oosterhout JJ, Hosseinipour M, Muula AS, Amberbir A, Wroe E, Berman J, et al. The Malawi NCD BRITE Consortium: Building Research Capacity, Implementation, and Translation Expertise for Noncommunicable Diseases. Glob Heart 2019;14:149–54.

21. Creswell JW. Steps in Conducting a Scholarly Mixed Methods Study. DBER Speak Ser 2013:54.

22. Muula AS. Case for clinical officers and medical assistants in Malawi. Croat Med J 2009;50:77.

23. Makwero MT. Delivery of primary health care in Malawi. African J Prim Heal Care Fam Med 2018;10:1–3.

24. World Health Organization. Sample Questionnaire for Rapid Assessment. Geneva, Switzerland: World Health Organization; 2012.

25. Birt L, Scott S, Cavers D, Campbell C, Walter F. Member checking: a tool to enhance trustworthiness or merely a nod to validation? Qual Health Res 2016;26:1802–11. doi:10.1177/1049732316654870.

26. Ritchie J, Spencer L. Qualitative data analysis for applied policy research. Anal. Qual. data, Routledge; 2002, p. 187–208.

27. Guetterman TC, Fetters MD, Creswell JW. Integrating quantitative and qualitative results in health science mixed methods research through joint displays. Ann Fam Med 2015;13:554–61.

28. World Health Organization. Prevention and control of noncommunicable diseases: guidelines for primary health care in low resource settings. Geneva, Switzerland: World Health Organization; 2012.

29. Ingenhoff R, Munana R, Weswa I, Gaal J, Sekitoleko I, Mutabazi H, et al. Principles for task shifting hypertension and diabetes screening and referral: a qualitative study exploring patient, community health worker and healthcare professional perceptions in rural Uganda. BMC Public Health 2023;23:881.

30. Kapongo RY, Lulebo AM, Mafuta EM, Mutombo PB, Dimbelolo JCM, Bieleli IE. Assessment of health service delivery capacities, health providers' knowledge and practices related to type 2 diabetes care in Kinshasa primary healthcare network facilities, Democratic Republic of the Congo. BMC Health Serv Res 2015;15:9.

31. Kengne AP, Mayosi BM. Readiness of the primary care system for non-communicable diseases in sub-Saharan Africa. Lancet Glob Heal 2014;2:e247–8. doi:10.1016/S2214-109X(14)70212-8.

32. Jaca A, Malinga T, Iwu-Jaja CJ, Nnaji CA, Okeibunor JC, Kamuya D, et al. Strengthening the health system as a strategy to achieving a universal health coverage in underprivileged communities in Africa: a scoping review. Int J Environ Res Public Health 2022;19:587.

33. Atun R, Davies JI, Gale EAM, Bärnighausen T, Beran D, Kengne AP, et al. Diabetes in sub-Saharan Africa: from clinical care to health policy. Lancet Diabetes Endocrinol 2017;5. doi:10.1016/S2213-8587(17)30181-X.

34. World Health Organization. Assessing national capacity for the prevention and control of noncommunicable diseases: report of the 2019 global survey. Geneva: 2020. doi:Licence: CC BY-NC-SA 3.0 IGO.

35. Malawi Government. Health Sector Strategic Plan II (2017-2022) 2017.

36. Malawi government. Malawi Standard Treatment Guidelines. 5th ed. Lilongwe, Malawi: Ministry of Health; 2015.

37. Sloan D, Phiri T, Zijlstra E. The Clinical Book. Blantyre, Malawi: College of Medicine, Malawi; 2013.

38. Mathanga DP, Walker ED, Wilson ML, Ali D, Taylor TE, Laufer MK. Malaria control in Malawi: current status and directions for the future. Acta Trop 2012;121:212–7.

39. Kanyerere H, Mganga A, Harries AD, Tayler-Smith K, Jahn A, Chimbwandira FM, et al. Decline in national tuberculosis notifications with national scale-up of antiretroviral therapy in Malawi. Public Heal Action 2014;4:113–5.

40. Harries AD, Ford N, Jahn A, Schouten EJ, Libamba E, Chimbwandira F, et al. Act local, think global: how the Malawi experience of scaling up antiretroviral treatment has informed global policy. BMC Public Health 2016;16:938.

41. King MF, Bruner GC. Social desirability bias: a neglected aspect of validity testing. Psychol Mark 2000;17:79–103. doi:10.1002/(SICI)1520-6793(200002)17.

figure
Click here to access/download;Figure;Banda et al_Fig1.png

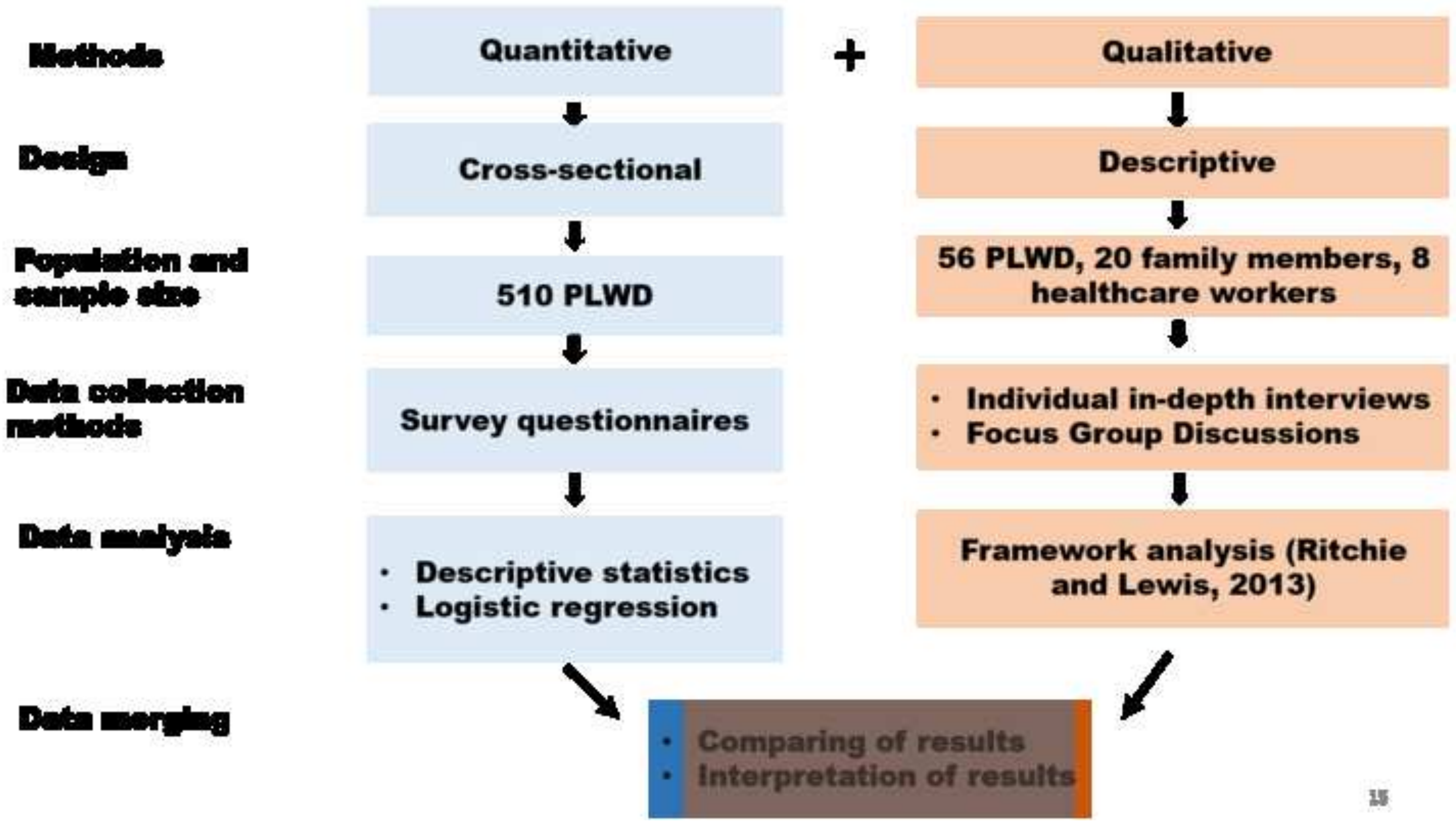

Click here to access/download
Supporting Information
Appendix 2_INDEPTH INTERVIEW GUIDE FOR KWY INFORMANT.docx

Click here to access/download
Supporting Information
Appendix 1- Needs Assessment Tool.docx
