# Supplementary material for: Primary health care preparedness to integrate diabetes care in Blantyre, Malawi: A mixed methods study
Source: PLoS One. 2024 May 21;19(5):e0303030. doi: 10.1371/journal.pone.0303030 (PMC11108178; doi:10.1371/journal.pone.0303030)
Supplement: Attachment — Submitted filename: Response to reviwers.docx [file pone.0303030.s004.docx]

Dear Dr Kelly,

Thank you for the feedback to our manuscript titled “Primary healthcare preparedness to integrate diabetes care in Blantyre, Malawi: a mixed methods study” submitted to PLOS ONE. We have carefully addressed each of the comments raised as below. Our response to the comments are in red for ease of identification.

1. When submitting your revision, we need you to address these additional requirements.

Please ensure that your manuscript meets PLOS ONE's style requirements, including those for file naming. The PLOS ONE style templates can be found at

<https://journals.plos.org/plosone/s/file?id=wjVg/PLOSOne_formatting_sample_main_body.pdf> and

<https://journals.plos.org/plosone/s/file?id=ba62/PLOSOne_formatting_sample_title_authors_affiliations.pdf>

Thank you. The manuscript has been revised to meet the PLOS ONE’s style as follows:

- the document has been changed to double space paragraph format

- initials of the corresponding author have been listed in parentheses after the email address

- symbols have been added to authors with equal contribution

- Figure 1 has been saved as Fig .tif

2. We note that the grant information you provided in the ‘Funding Information’ and ‘Financial Disclosure’ sections do not match.

- apologies for the confusion. The funding information has been updated and should read:

Research reported in this publication was supported by the National Heart, Lung, And Blood Institute of the National Institutes of Health under Award Number U24HL136791. The content is solely the responsibility of the authors and does not necessarily represent the official views of the National Institutes of Health.

CKB was supported by the Consortium for Advanced Research Training in Africa (CARTA). CARTA is jointly led by the African Population and Health Research Center and the University of the Witwatersrand and funded by the Carnegie Corporation of New York (Grant No. G-19-57145), Sida (Grant No:54100113), Uppsala Monitoring Center, Norwegian Agency for Development Cooperation (Norad), and by the Wellcome Trust [reference no. 107768/Z/15/Z] and the UK Foreign, Commonwealth & Development Office, with support from the Developing Excellence in Leadership, Training and Science in Africa (DELTAS Africa) programme. The statements made and views expressed are solely the responsibility of the Fellow. For the purpose of open access, the author has applied a CC BY public copyright licence to any Author Accepted Manuscript version arising from this submission."

There was no additional external funding received for this study.

When you resubmit, please ensure that you provide the correct grant numbers for the awards you received for your study in the ‘Funding Information’ section.

- this is noted

3. Thank you for stating in your Funding Statement:

Research reported in this publication was supported by the National Heart, Lung, And Blood Institute of the National Institutes of Health under Award Number U24HL136791. The content is solely the responsibility of the authors and does not necessarily represent the official views of the National Institutes of Health.

This research was also supported by the Consortium for Advanced Research Training in Africa (CARTA). CARTA is jointly led by the African Population and Health Research Center and the University of the Witwatersrand and funded by the Carnegie Corporation of New York (Grant No--B 8606.R02), Sida (Grant No:54100029), the DELTAS Africa Initiative (Grant No: 107768/Z/15/Z). The DELTAS Africa Initiative is an independent funding scheme of the African Academy of Sciences (AAS)’s Alliance for Accelerating Excellence in Science in Africa (AESA) and supported by the New Partnership for Africa’s Development Planning and Coordinating Agency (NEPAD Agency) with funding from the Wellcome Trust (UK) (Grant No: 107768/Z/15/Z) and the UK government, the statements made and views expressed are solely the responsibility of the fellow.

Please provide an amended statement that declares all the funding or sources of support (whether external or internal to your organization) received during this study, as detailed online in our guide for authors at http://journals.plos.org/plosone/s/submit-now. Please also include the statement “There was no additional external funding received for this study.” in your updated Funding Statement.

Please include your amended Funding Statement within your cover letter. We will change the online submission form on your behalf.

The funding statement has been updated and included the statement: “There was no additional external funding received for this study.”

4. Thank you for stating the following in the Acknowledgments Section of your manuscript:

Research reported in this publication was supported by the National Heart, Lung, And Blood Institute of the National Institutes of Health under Award Number U24HL136791. The content is solely the responsibility of the authors and does not necessarily represent the official views of the National Institutes of Health.

This research was also supported by the Consortium for Advanced Research Training in Africa (CARTA). CARTA is jointly led by the African Population and Health Research Center and the University of the Witwatersrand and funded by the Carnegie Corporation of New York (Grant No--B 8606.R02), Sida (Grant No:54100029), the DELTAS Africa Initiative (Grant No: 107768/Z/15/Z). The DELTAS Africa Initiative is an independent funding scheme of the African Academy of Sciences (AAS)’s Alliance for Accelerating Excellence in Science in Africa (AESA) and supported by the New Partnership for Africa’s Development Planning and Coordinating Agency (NEPAD Agency) with funding from the Wellcome Trust (UK) (Grant No: 107768/Z/15/Z) and the UK government, the statements made and views expressed are solely the responsibility of the fellow.

We note that you have provided funding information that is not currently declared in your Funding Statement. However, funding information should not appear in the Acknowledgments section or other areas of your manuscript. We will only publish funding information present in the Funding Statement section of the online submission form.

Please remove any funding-related text from the manuscript and let us know how you would like to update your Funding Statement. Currently, your Funding Statement reads as follows:

Research reported in this publication was supported by the National Heart, Lung, And Blood Institute of the National Institutes of Health under Award Number U24HL136791. The content is solely the responsibility of the authors and does not necessarily represent the official views of the National Institutes of Health.

This research was also supported by the Consortium for Advanced Research Training in Africa (CARTA). CARTA is jointly led by the African Population and Health Research Center and the University of the Witwatersrand and funded by the Carnegie Corporation of New York (Grant No--B 8606.R02), Sida (Grant No:54100029), the DELTAS Africa Initiative (Grant No: 107768/Z/15/Z). The DELTAS Africa Initiative is an independent funding scheme of the African Academy of Sciences (AAS)’s Alliance for Accelerating Excellence in Science in Africa (AESA) and supported by the New Partnership for Africa’s Development Planning and Coordinating Agency (NEPAD Agency) with funding from the Wellcome Trust (UK) (Grant No: 107768/Z/15/Z) and the UK government, the statements made and views expressed are solely the responsibility of the fellow.

Please include your amended statements within your cover letter; we will change the online submission form on your behalf.

Apologies for that, the funding statement has been removed from the acknowledgements section. The acknowledgements text now reads:

We would like to thank the Blantyre DHO management and staff for their support and assistance in the needs assessment.

5. Please amend either the title on the online submission form (via Edit Submission) or the title in the manuscript so that they are identical.

the title in the manuscript has been edited to match that in the online submission and reads:

Primary healthcare preparedness to integrate diabetes care in Blantyre, Malawi: a mixed methods study

6. Please upload a new copy of Figure 1 as the detail is not clear. Please follow the link for more information: https://blogs.plos.org/plos/2019/06/looking-good-tips-for-creating-your-plos-figures-graphics/" https://blogs.plos.org/plos/2019/06/looking-good-tips-for-creating-your-plos-figures-graphics/

A new copy of Figure 1 has been created and uploaded

7. Please review your reference list to ensure that it is complete and correct. If you have cited papers that have been retracted, please include the rationale for doing so in the manuscript text, or remove these references and replace them with relevant current references. Any changes to the reference list should be mentioned in the rebuttal letter that accompanies your revised manuscript. If you need to cite a retracted article, indicate the article’s retracted status in the References list and also include a citation and full reference for the retraction notice.

The reference list has been reviewed and we did not find any papers that were retracted.

The following references have been added to the manuscript after revisions from the reviewer:

4.	Timpel P, Lang C, Wens J, Contel JC, Schwarz PEH, Group MCS. The Manage Care Model–Developing an Evidence-Based and Expert-Driven Chronic Care Management Model for Patients with Diabetes. Int J Integr Care 2020;20.

5.	Wroe EB, Kalanga N, Dunbar EL, Nazimera L, Price NF, Shah A, et al. Expanding access to non-communicable disease care in rural Malawi: outcomes from a retrospective cohort in an integrated NCD–HIV model. BMJ Open 2020;10:e036836.

24.	International Diabetes Federation. IDF Diabetes Atlas 10th Edition - Malawi 2021. https://diabetesatlas.org/data/en/country/119/mw.html (accessed February 19, 2024).

28.	Conroy R. The RCSI Sample size handbook. 2018. doi:10.13140/RG.2.2.30497.51043.

35.	Flood D, Geldsetzer P, Agoudavi K, Aryal KK, Brant LCC, Brian G, et al. Rural-Urban Differences in Diabetes Care and Control in 42 Low- and Middle-Income Countries: A Cross-sectional Study of Nationally Representative Individual-Level Data. Diabetes Care 2022;45:1961–70. doi:10.2337/dc21-2342.

The following reference has been removed from the list following revisions:

Jaca A, Malinga T, Iwu-Jaja CJ, Nnaji CA, Okeibunor JC, Kamuya D, Wiysonge CS. Strengthening the health system as a strategy to achieving a universal health coverage in underprivileged communities in Africa: a scoping review. International journal of environmental research and public health. 2022 Jan 5;19(1):587.

[Note: HTML markup is below. Please do not edit.]

Reviewers' comments:

Reviewer's Responses to Questions

Comments to the Author

1. Is the manuscript technically sound, and do the data support the conclusions?

The manuscript must describe a technically sound piece of scientific research with data that supports the conclusions. Experiments must have been conducted rigorously, with appropriate controls, replication, and sample sizes. The conclusions must be drawn appropriately based on the data presented.

Reviewer #1: Yes

2. Has the statistical analysis been performed appropriately and rigorously?

Reviewer #1: Yes

3. Have the authors made all data underlying the findings in their manuscript fully available?

The PLOS Data policy requires authors to make all data underlying the findings described in their manuscript fully available without restriction, with rare exception (please refer to the Data Availability Statement in the manuscript PDF file). The data should be provided as part of the manuscript or its supporting information, or deposited to a public repository. For example, in addition to summary statistics, the data points behind means, medians and variance measures should be available. If there are restrictions on publicly sharing data—e.g. participant privacy or use of data from a third party—those must be specified.

Reviewer #1: Yes

4. Is the manuscript presented in an intelligible fashion and written in standard English?

PLOS ONE does not copyedit accepted manuscripts, so the language in submitted articles must be clear, correct, and unambiguous. Any typographical or grammatical errors should be corrected at revision, so please note any specific errors here.

Reviewer #1: Yes

5. Review Comments to the Author

Please use the space provided to explain your answers to the questions above. You may also include additional comments for the author, including concerns about dual publication, research ethics, or publication ethics. (Please upload your review as an attachment if it exceeds 20,000 characters)

Reviewer #1: A good and informative paper. However, the authors need to give more details in the methodology and result sections. Specifically, there is no specific formula quoted in finding the sample size of 14 in the quantitative data collection. In the results section, there are two tables where it would be useful for the authors to provide national standard figures for staff and equipment. There are certain paragraphs in the the discussion that would be better off rephrased. Further, there's little discussion on the differences between the urban and rural facilities and how the differences impact utilization in the 2 areas. More details of my comments are provided in the paper against specific sections.

- an explanation on the sample size has been given as follows: Fourteen facilities (seven urban and seven rural) from Blantyre DHO were included in the assessment. Given that urban Blantyre had only 7 facilities, we decided to select all of them to ensure good coverage [28]. We then randomly selected 7 health facilities in rural Blantyre to ensure a balance between rural and urban thus giving a total of 14 facilities. We did not perform a sample size calculation because of the small number of the sampling units (i.e. health centers).
- paragraphs have been rephrased as suggested all through the paper
- the discussion section has been redone to show the differences between urban and rural facilities and how the difference impact utilization
- All comments provided in paper against specific sections have been addressed and are shown in the paper with track changes.

6. PLOS authors have the option to publish the peer review history of their article (what does this mean?). If published, this will include your full peer review and any attached files.

If you choose “no”, your identity will remain anonymous but your review may still be made public.

Do you want your identity to be public for this peer review? For information about this choice, including consent withdrawal, please see our Privacy Policy.

Reviewer #1: No

Thank you,

Chimwemwe Kwanjo Banda - Corresponding author

20^th^ February, 2024
